# Syntaxin-1 is necessary for UNC5A-C/Netrin-1-dependent macropinocytosis and chemorepulsion

**DOI:** 10.3389/fnmol.2023.1253954

**Published:** 2023-09-27

**Authors:** Ramón Martínez-Mármol, Ashraf Muhaisen, Tiziana Cotrufo, Cristina Roselló-Busquets, Oriol Ros, Marc Hernaiz-Llorens, Francesc Pérez-Branguli, Rosa Maria Andrés, Antoni Parcerisas, Marta Pascual, Fausto Ulloa, Eduardo Soriano

**Affiliations:** ^1^Department of Cell Biology, Physiology and Immunology, Faculty of Biology and Institute of Neurosciences, Universitat de Barcelona (UB), Barcelona, Spain; ^2^Clem Jones Centre for Ageing Dementia Research, Queensland Brain Institute, The University of Queensland, Brisbane, QLD, Australia; ^3^Centro de Investigación Biomédica en Red Sobre Enfermedades Neurodegenerativas (CIBERNED-CIBER), ISCIII, Madrid, Spain; ^4^IZKF Junior Research Group and BMBF Research Group Neuroscience, IZKF, Friedrich-Alexander-Universitaet Erlangen-Nuernberg, Erlangen, Germany; ^5^Tissue Repair and Regeneration Laboratory (TR2Lab), Institut de Recerca i Innovació en Ciències de la Vida i de la Salut a la Catalunya Central (IRIS-CC), Vic, Spain; ^6^Biosciences Department, Faculty of Sciences, Technology and Engineerings, University of Vic - Central University of Catalonia (UVic-UCC), Vic, Spain

**Keywords:** axon guidance, macropinocytosis, Netrin-1, syntaxin-1, UNC5 receptors

## Abstract

**Introduction:**

Brain connectivity requires correct axonal guidance to drive axons to their appropriate targets. This process is orchestrated by guidance cues that exert attraction or repulsion to developing axons. However, the intricacies of the cellular machinery responsible for the correct response of growth cones are just being unveiled. Netrin-1 is a bifunctional molecule involved in axon pathfinding and cell migration that induces repulsion during postnatal cerebellar development. This process is mediated by UNC5 homolog receptors located on external granule layer (EGL) tracts.

**Methods:**

Biochemical, imaging and cell biology techniques, as well as syntaxin-1A/B (Stx1A/B) knock-out mice were used in primary cultures and brain explants.

**Results and discussion:**

Here, we demonstrate that this response is characterized by enhanced membrane internalization through macropinocytosis, but not clathrin-mediated endocytosis. We show that UNC5A, UNC5B, and UNC5C receptors form a protein complex with the t-SNARE syntaxin-1. By combining botulinum neurotoxins, an shRNA knock-down strategy and Stx1 knock-out mice, we demonstrate that this SNARE protein is required for Netrin1-induced macropinocytosis and chemorepulsion, suggesting that Stx1 is crucial in regulating Netrin-1-mediated axonal guidance.

## Introduction

During the development of the nervous system, migrating cells and axonal growth cones respond to attractive and repellent guidance cues. Netrin-1 belongs to a family of laminin-related secreted proteins that act as bifunctional guidance factors, generating chemoattractive or chemorepulsive responses. Specific receptors on the surface of the growth cones determine the response to Netrin-1. Netrin-1-induced axon attraction is mediated by Deleted in Colorectal Cancer (DCC) receptors (Keino-Masu et al., 1996), Neogenin/DCC like molecule (De Vries and Cooper, 2008) and Down Syndrome Cell Adhesion Molecule (DSCAM) receptors (Ly et al., 2008). The Uncoordinated movement 5 family of receptors (UNC5A, B, C and D) mediates repulsion (Hedgecock et al., 1990; Keleman and Dickson, 2001). The repulsive action of Netrin-1 requires axonal expression of either an UNC5 homolog alone or a complex of UNC5 and either DCC or DSCAM receptors (Hong et al., 1999; Purohit et al., 2012). Interestingly, recent studies pointed to a combinatorial relationship between different receptors to control a particular axon response to the cue (Muramatsu et al., 2010; Boyer and Gupton, 2018).

Once axons reach their final destination, neuronal communication starts by releasing neurotransmitters contained in synaptic vesicles. Synaptic vesicle exocytosis occurs through the assembly of the SNARE (Soluble NSF-Attachment protein Receptors) protein complex. It requires association of the plasma membrane t-SNAREs syntaxin-1 (Stx1) and SNAP25 with the vesicle v-SNARE VAMP2/Synaptobrevin 2 (Jahn and Scheller, 2006). In addition, other synaptic proteins, including Synaptotagmins, Munc-18 and Complexin regulate the exocytotic cycle of synaptic vesicles (Brunger et al., 2019). Interestingly, many proteins involved in synaptic exocytosis and endocytosis are highly expressed during development and are enriched in growth cones (Urbina and Gupton, 2020). A number of studies have supported the notion that axonal guidance mediated by extracellular cues may rely on the control of exocytotic and endocytic events at selected regions of the growth cone (Tojima et al., 2007, 2010; Hines et al., 2010; Cotrufo et al., 2011; Zylbersztejn et al., 2012).

During chemoattraction, Netrin-1 triggers the recruitment of its receptor DCC to the plasma membrane and increases growth cone exocytosis (Bouchard et al., 2004, 2008; Matsumoto and Nagashima, 2010). Moreover, Stx1 directly interacts with DCC and is required for Netrin-1-dependent chemoattraction of axons and migrating neurons, both *in vitro* and *in vivo* (Cotrufo et al., 2011, 2012; Barrecheguren et al., 2017). Together these data indicate a tight cross-talk between chemoattractive guidance cue signaling pathways and proteins that regulate exocytosis within growth cones.

In contrast, the relationship between chemorepellent cues and proteins that regulate membrane turnover needs to be better understood. Clathrin-dependent endocytosis drives repulsive growth cone responses to Semaphorin 3A (Tojima et al., 2010). In dorsal root ganglion, retinal ganglion cells and commissural growth cones, Ephrin-A2, Sonic hedgehog (Shh) and Slit2 stimulate a specific type of clathrin-independent endocytosis of large structures known as macropinocytosis (Jurney et al., 2002; Kabayama et al., 2009; Guo et al., 2012; Ros et al., 2018). Macropinocytosis is a particular form of endocytosis characterized by the non-specific, clathrin-or-caveolin-independent formation of large endocytic vacuoles (macropinosomes) that internalize large amounts of liquid, solute, and membrane (Ferreira and Boucrot, 2018; Lin et al., 2020). The vesicle SNARE VAMP-2 mediates the sorting of Neuropilin-1/Plexin-A1 receptors, which is required for repulsion by Semaphorin 3A (Zylbersztejn et al., 2012). Overall, these studies led to the assumption that membrane remodeling, acting in coordination with F-actin cytoskeletal reorganization, may contribute to growth cone steering by chemorepellent guidance cues (Gallo and Letourneau, 2004; Piper et al., 2006). Nevertheless, the mechanistic role of SNARE proteins in these processes remains unclear.

Here we investigate whether Netrin-1-dependent growth cone collapse and chemorepulsion are associated with enhanced membrane internalization. We show that both Netrin-1-induced collapse and axon chemorepulsion are associated with macropinocytosis. Furthermore, we demonstrate that the SNARE protein Stx1 co-associates with the Netrin-1 receptors UNC5 and that Stx1 function is required for Netrin-1-induced membrane macropinocytosis and repulsion. Overall, our results underscore a novel Stx1-dependent signaling pathway necessary for membrane internalization in Netrin-1-mediated chemorepulsion.

## Materials and methods

### Plasmids

Full-length Stx1A and Stx1A-GFP were created as described previously (Cotrufo et al., 2011). UNC5A-myc, UNC5B-myc and UNC5C-myc were a gift from Prof. Lindsay Hinck (University of California, Santa Cruz, Santa Cruz, CA, USA), pEGFP-C1 (Clontech), pcDNA3.1 (Invitrogen, Thermo Fisher Scientific), GFP-EpsΔ95/295 vector was a gift from Dr. Francesc Tebar (University of Barcelona, Spain). For shRNA experiments, the pLVTHM plasmid was kindly provided by Prof. Didier Trono (École polytechnique fédérale de Lausanne, Lausanne), including specific oligonucleotides for Stx1A and Stx1B sequence: gatcccc CCAGAGGCAGCTGGAGATCACttcaagagaGGTCTCCGTCGAC CTCTAGTGttttt (Forward), and agctaaaaaCCAGAGGCA GCTGGAGATCACtctcttgaaGGTCTCCGTCGACCTCTAGTGggg (Reverse).

### Heterologous cell cultures

HEK-293T cells were maintained in DMEM (11995065; GIBCO-Thermo Fisher Scientific) medium supplemented with 10% fetal bovine serum (FBS; 26140079; GIBCO-Thermo Fisher Scientific), 1% GlutaMAX (35050061; GIBCO-Thermo Fisher Scientific) and 1% penicillin/streptomycin (15140122; GIBCO-Thermo Fisher Scientific). PC12 cells were maintained in DMEM containing 1% GlutaMAX, 5% FBS, 5% horse serum (HS; 26050-088; GIBCO-Thermo Fisher Scientific), and 1% penicillin/streptomycin.

### Primary neuronal cultures

Primary cultures and explants of cerebellar EGL neurons were prepared from P3 to P5 postnatal CD1 strain mice (Charles River). Animals were sacrificed by decapitation in accordance with institutional and governmental ethical guidelines and regulations. All the experiments using animals were performed in accordance with the European Community Council directive and the National Institutes of Health guidelines for the care and use of laboratory animals. Experiments were also approved by the ethical committee from the Generalitat of Catalonia. Postnatal cerebellums were isolated, mechanically disaggregated and trypsinized as previously described (Rosello-Busquets et al., 2019; Hernaiz-Llorens et al., 2020). Briefly, after centrifugation, neurons were resuspended in 2 mL of DMEM medium and EGL neurons were isolated by centrifugation (3000 rpm, 10 min at 4°C) in a discontinuous percoll gradient (35 and 60% of percoll). After washing with PBS, EGL neurons were plated on poly-D-lysine pre-coated dishes in DMEM, 1% penicillin/streptomycin, 1% glutamine, 4.5% D-(+)-glucose (G-8769; Sigma), 5% HS and 10% FBS. This medium was maintained for 24 h and then changed to a medium where HS and FBS were replaced by 2% B27 (17504001; GIBCO-Thermo Fisher Scientific) and 1% N2 (11520536; GIBCO-Thermo Fisher Scientific). Neurons were cultured for 72 h (3DIV).

To isolate and culture EGL explants, cerebellums from P3 to P5 mice were isolated and chopped in to 300 μm slices. Selected slices were further dissected using fine tungsten needles to extract small tissue pieces from the EGL. Explants were carefully placed inside a 3D collagen matrix on poly-D-lysine pre-coated dishes, prepared as previously described (Gil and Del Rio, 2019). Explants were cultured for 48 h (2DIV) in DMEM, 1% penicillin/streptomycin, 1% glutamine, 4.5% D-(+)-glucose, 2% B27.

### Transfection of heterologous cells and primary neurons, and electroporation of explants

To downregulate the expression of both Stx1A and Stx1B, a shRNA directed both Stx1A and Stx1B was used to downregulate the expression levels of the two paralogs, and a scramble shRNA was used as negative control [21]. The coding mRNA sequence for both *Stx1A* and *Stx1B* in mouse (*mus musculus*) and rat (*rattus norvegicus*) share 97 and 96% identity, respectively, and are identical in the shRNA-recognized region. One day before transfection, cells were counted and plated in 100 mm dishes, to have a 70% of confluence on the day of transfection. HEK-293T or PC12 cells were transfected with Lipofectamine 2000 (Thermo Fisher Scientific) following the manufacturer’s instructions.

After 2DIV, neuronal cultures were transfected using Lipofectamine 2000. Before transfection, 3/5 of the medium was removed and kept aside to be returned later. For each 35 mm plate, 4 μg of DNA and 8 μL of Lipofectamine 2000 were used. The final mixture of DNA-Lipofectamine 2000 was carefully added to the cultures and further incubated at 37°C in 5% CO_2_ for 60 min. The transfection medium containing DNA and Lipofectamine 2000 was finally replaced by the medium removed at the beginning. An equal amount of freshly prepared medium was added. Cultures were incubated until the following day.

Explants were electroporated using the Invitrogen Neon system. Briefly, dissected explants were washed three times in PBS and resuspended in buffer R containing 2 to 4 μg of the DNA to electroporate. The conditions for the electroporation were voltage: 500 V; width: 50 ms; 5 pulses. Explants were immediately washed once in Neurobasal medium and then mounted in a 3D collagen matrix.

### Immunocytochemistry

PC12 cells and primary neurons were fixed with a solution of 4% paraformaldehyde (PFA) in PBS for 10 min at room temperature. Neuronal explants were fixed by incubation with the same solution for 30 min at room temperature. They were rinsed with PBS, and permeabilized with a solution of 0.1% Triton-X-100 in PBS for 10, or 30 min for the explants. Cells were then washed with PBS and incubated in blocking solution (10% HS in PBS) for 1 h at room temperature, or 3 h for the explants. After blocking, the cells were incubated with the respective primary antibodies diluted in blocking solution for 2 h at room temperature or overnight at 4°C for the explants. Cells and explants were washed with PBS and incubated in the secondary antibody solution (PBS 1x with 10% HS) for 1 h at room temperature, or 3 h for the explants. Finally, cells and explants were washed and mounted in Mowiol (Sigma-Aldrich) for imaging.

Cholera toxin B-subunit (CTxB) Alexa Fluor 488-conjugated (C22841; Invitrogen-Thermo Fisher Scientific). Colocalization of UNC5C receptor with Stx1 and the lipid raft membrane marker CTxB was quantified in growth cones manually selected using Fiji wand tool, and performing thresholding of the corresponding channels (UNC5C, Stx1 or CTxB) to obtain binary masks that were overlapped. Colocalization was considered as the fraction of UNC5C area that overlaps with Stx1 or the raft marker.

### Immunoprecipitation assays and immunoblots

Cells were lysed in lysis buffer (50 mM Tris pH 7.2, 150 mM NaCl, 5 mM EDTA, 1% Triton, 10% glycerol, 10 μg/ml Leupeptin, 10 μg/ml Aprotinin, 1 mM PMSF). For the immunoprecipitation assays, 50–500 μg of total protein per brain tissue sample or transfected HEK-293T cell homogenates were used. Briefly, samples were first pre-cleared with 30 μL of protein G-sepharose beads (P3391, Sigma). Additional protein G-sepharose beads was blocked in 5% bovine serum albumin (BSA) and stored at 4°C. After 1 h of pre-clearing and blocking, samples were incubated overnight at 4°C with mouse anti-myc (Sigma-Aldrich; 1/5000), mouse anti-GFP (11814460001, Sigma-Aldrich; 1/500) or mouse anti-Sytx1 (HPC-1 clone; Sigma-Aldrich; 1:500) antibodies. Negative controls were performed by immunoprecipitation with Sepharose beads alone or with the non-interacting antibody rabbit anti-Egr1 (Invitrogen Thermo Fisher-Scientific; 1:50) (Cotrufo et al., 2011). Blocked protein G-sepharose beads was added and incubated for 2 h at 4°C. After five washes with wash buffer (10 mM Tris pH 7, 500 mM NaCl, 1 mM EDTA, 1 mM EGTA, 1% Triton X-100, 0.5% NP-40), SDS-sample buffer was added to the beads and the proteins were analyzed by SDS-PAGE and Western Blot. Proteins were transferred onto nitrocellulose membranes, which were blocked with 5% non-fat dry milk in Tris-HCl buffered saline (TBS) containing 0.1% Tween 20, and incubated overnight at 4°C with goat anti-UNC5C (ab106949; Abcam; 1/500), mouse anti-Sytx1 (1:3000), rabbit anti-myc (1:1000), or rabbit anti-GFP (A11122; Invitrogen-Thermo Fisher Scientific; 1:1000) antibodies. After incubation with the appropriate HRP-conjugated secondary antibodies, blots were developed following the ECL method (Amersham Pharmacia Biotech). To separate the Sytx1A and Stx1B isoforms, brain protein samples were analyzed by Tris-urea/SDS-PAGE (18% acrylamide, 6 M urea, 750 mM Tris-HC1, pH 8.85, 50 mM NaCI, 0.1% SDS). The resulting bands were manually selected and quantified using ImageJ.

### Growth cone collapse and vesicle internalization experiments

External granule layer primary neurones were cultured for 3DIV and then treated with specific reagents or toxins, or the respective controls (DMSO or PBS), which were included 10 to 30 min before the addition of Netrin-1, and maintained during the whole incubation. 5-(N-ethyl-N-isopropyl) amiloride (EIPA) was purchased from SIGMA-MERCK, and botulinum neurotoxin type A (BoNT/A) and type C1 (BoNT/C1) were purchased from Metabiologics, INC. In growth cone collapse experiments, neurons were then incubated with Netrin-1 (300 ng/mL) or control (BSA 0.1%) for 45 min at 37°C in DMEM containing 1% glutamine and 4.5% D-(+)-glucose. After this incubation, neurons were fixed with 4% PFA and permeabilized with PBS-Triton-X-100 (0.1%). Finally, actin filaments were stained by incubation with phalloidin-TRITC (1 μM) for 30 min. Cells were mounted on Mowiol and used for imaging. Actin staining was used to identify growth cones, which were outlined based on differential staining for actin in this compartment with respect to the adjacent axon. When actin was not stained, growth cones were outlined based on their WGA-TRITC staining that highlighted their surface. An intensity threshold mask was created using Fiji (Schneider et al., 2012) and the growth cone perimeter was selected using the wand tool. Collapsing growth cones were manually identified based on their morphology. In contrast to normal extended growth cones with evident filopodia and lamellipodia, collapsing growth cones lose their spread morphology, rapidly (within minutes) acquiring a shriveled, round-tipped pencil-like shape largely devoid of lamellae or filopodia (Luo et al., 1993; Goshima et al., 1995). The percentage of growth cones containing macropinocytic (dextran-positive) endosomes, and the percentage of collapsing growth cones was calculated and plotted to evaluate the degree of growth cone collapse and macropinocytosis.

### Repulsion experiments

During the plating procedure, explants embedded into the 3D collagen matrix were confronted at a distance of 150–300 μm with aggregates of HEK-293T cells stably expressing Netrin-1 (Cotrufo et al., 2011), or transiently transfected with semaphorin 3A or semaphorin 3F. Netrin-1 expression in stable cells was regularly checked by western blot (data not shown). If required, explants were treated with EIPA, DMSO (control), or with specific BoNTs (25 nM BoNT/A or 15 nM BoNT/C1) by adding them to the medium 3 to 4 h after being cultured. After 48 h (2DIV) of culture, explants were fixed, and immunocytochemistry against βIII-tubulin and GFP was performed. Repulsion was analyzed by measuring the proximal/distal (P/D) ratio as previously described (Gil and Del Rio, 2019; Hernaiz-Llorens et al., 2020). Briefly, the P/D ratio is the fraction of axons growing in the proximal quadrant of the explant (closer to the aggregate of HEK-293T cells) vs. those growing in the distal quadrant. A ratio close to 1 indicates a radial growth pattern, below 1 indicates chemorepulsion and above 1 indicates chemoattraction. Only the GFP-positive axons were used for quantification in experiments with explants containing neurons expressing GFP.

### Statistics

Results were analyzed statistically using GraphPad Prism software (GraphPad Software, Inc). The D’Agostino and Pearson test was used to test for normality. The unpaired two-tailed Student’s *t*-test was used to compare two groups. For datasets comparing more than two groups, ANOVA followed by the Dunn test, Sidak’s or the Dunnet test corrections for multiple comparisons was used. Statistical comparisons were performed on a per-explant or per-experiment basis. If the number of elements analyzed for each condition was above 15, it is indicated within each bar. If the number was below 15, each value is plotted. When the comparison is performed on a per-experiment basis, individual data points are shown as the number is <15. Each of the experiments analyzed a large number of individual cones/axons, as stated in the corresponding figure legends. The neurons analyzed were randomly selected within the dishes and blindly analyzed, and were collected from at least three independent experiments. Values are represented as the mean ± SEM. The tests used are indicated in the respective figure legends. A *p*-value below 0.05 was accepted as significant. “ns” means non-significant.

## Results

### UNC5 receptors co-associate with Stx1 both *in vitro* and *in vivo*

**FIGURE 1:**
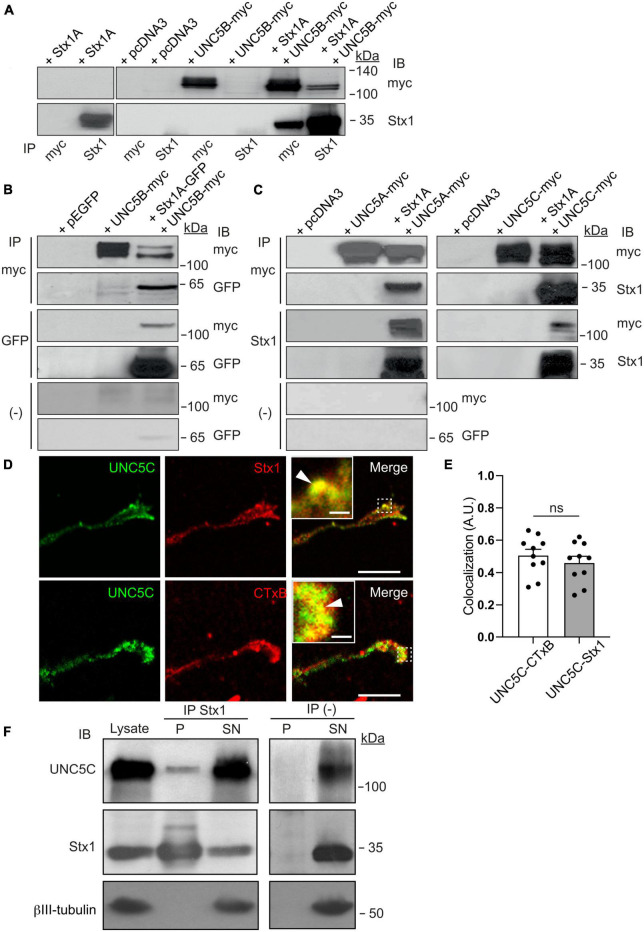
Stx1 interacts with UNC5 receptors. **(A)** HEK-293T cells were transfected with the indicated combination of plasmids (empty vector pcDNA3, Stx1A, UNC5B-myc, or both Stx1A and UNC5B-myc). Protein lysates were immunoprecipitated (IP) with anti-Stx1 or anti-myc antibodies. Co-immunoprecipitation of Stx1A and UNC5B was detected by immunoblotting (IB) using anti-Stx1 (Stx1) or anti-myc (myc) antibodies. **(B)** HEK-293T cells were transfected with the indicated combination of plasmids (empty vector pEGFP, Stx1A-GFP, UNC5B-myc, or both Stx1A-GFP and UNC5B-myc). Protein lysates were immunoprecipitated with anti-GFP (GFP), anti-myc antibodies or without antibodies [(−)]. Co-immunoprecipitation of Stx1A and UNC5B was detected by immunoblotting using anti-GFP or anti-myc antibodies. **(C)** HEK-293T cells were transfected with the indicated combination of plasmids (empty vector pcDNA3, Stx1A, UNC5A-myc, UNC5C-myc, Stx1A and UNC5A-myc, or Stx1A and UNC5C-myc). Protein lysates were immunoprecipitated with anti-Stx1, anti-myc antibodies or without antibodies. Co-immunoprecipitation of Stx1A and UNC5A or UNC5C was detected by immunoblotting using anti-Stx1 or anti-myc antibodies. **(D)** Representative confocal images of control (non-Netrin-1 treated) EGL growth cones immunostained against UNC5C and Stx1 or the lipid raft marker CTxB, showing colocalization of UNC5 with Sytx1 and its enrichment in lipid rafts. Insets in merge panels show higher magnification of colocalization regions (arrowheads). Scale bars represent 10 μm in images and 1 μm in inset. **(E)** Histogram showing a high degree of colocalization of UNC5 with Sytx1 and CTxB. **(F)** UNC5C immunoprecipitation with Stx1 from P4 cerebellar homogenates. Immunoblotting using anti-Stx1 antibody was used as a positive control, and anti-βIII-tubulin antibody was used as a loading control and as a negative immunoprecipitation control. Data in panel **(E)** represent mean ± SEM. Each data point plotted represents one neuron. Unpaired two-tailed Student’s *t*-test was used. ns stands for non-significant (*p* > 0.05); IB stands for immunoblotting; IP stands for immunoprecipitation.

We have previously demonstrated that the t-SNARE Stx1 interacts with the guidance receptor DCC (Cotrufo et al., 2011, 2012) and the tropomyosin-related kinase (TrkB) receptor (Fuschini et al., 2018), with these associations being necessary for the Netrin-1-dependent chemoattraction and neurotrophin-dependent outgrowth of axons, respectively. These results indicate that Stx1 regulates exocytosis and membrane retrieval in growth cones and is required for Netrin-1-induced chemotropic guidance *in vitro* and *in vivo* (Ros et al., 2015). On the other hand, it has also been suggested that SNARE proteins can be involved in certain types of endocytosis within synapses (Xu et al., 2013; Zhang et al., 2013), and that some SNAREs interact with proteins involved in endocytosis (Galas et al., 2000; Koo et al., 2011; Miller et al., 2011). In addition, we have found that Stx1 is required for the repulsion of commissural axons (Ros et al., 2018). Here, we first investigated whether Stx1 interacts with the chemorepulsive Netrin-1 receptors UNC5A, UNC5B, and UNC5C, which are essential in controlling the repulsive response of axonal tracts during cerebellar development (Alcantara et al., 2000; Kim and Ackerman, 2011). First, we tested this interaction using an *in vitro* system in which HEK-293T cells were co-transfected with Stx1A or Stx1A-EGFP, together with UNC5B-myc, and tested for co-immunoprecipitation (Figure 1). Our results revealed that immunoprecipitation with anti-myc antibodies led to Stx1 association (Figure 1A). The reverse immunoprecipitation with anti-Stx1 antibodies also yielded myc-tagged UNC5B (Figure 1A). This interaction was maintained using anti-GFP antibodies to pull down Stx1A-EGFP (Figure 1B). In similar co-immunoprecipitation experiments, co-transfecting UNC5A-myc or UNC5C-myc with Stx1A-EGFP revealed that Stx1 also co-associates with UNC5A and UNC5C receptors (Figure 1C). Furthermore, control immunoprecipitations with beads alone did not show co-association (Figures 1B, C). Lack of immunoprecipitation with irrelevant antibodies (anti-Egr1) further suggested that the observed interaction was specific (Supplementary Figure 1). Next, we asked whether Stx1 is associated with UNC5 receptors *in vivo*. Using cultured postnatal cerebellar EGL neurons, we found that both proteins partially colocalize in control (Netrin-1 untreated) growth cones (Figure 1D) to a similar extent to the colocalization with lipid rafts microdomains labeled with cholera toxin subunit B (CTxB) (Figure 1E, inset in Figure 1D) (Hernaiz-Llorens et al., 2020). Moreover, immunoprecipitation of postnatal day 4 (P4) cerebellar cortical tissue with Stx1 revealed interaction with UNC5C (Figure 1F). Finally, to investigate whether Stx1 interacts with other receptors that mediate chemorepulsion, we co-transfected HEK-293T cells with Stx1A-EGFP and Neuropilin-1-HA or Plexin-A1-VSV, the receptors for class III Semaphorins (Gil and Del Rio, 2019). Our results showed that Stx1 does not interact with these Semaphorin receptors (Supplementary Figure 2).

### Botulinum neurotoxin C1 decreases the Netrin-1-dependent chemorepulsion of EGL axons

**FIGURE 2:**
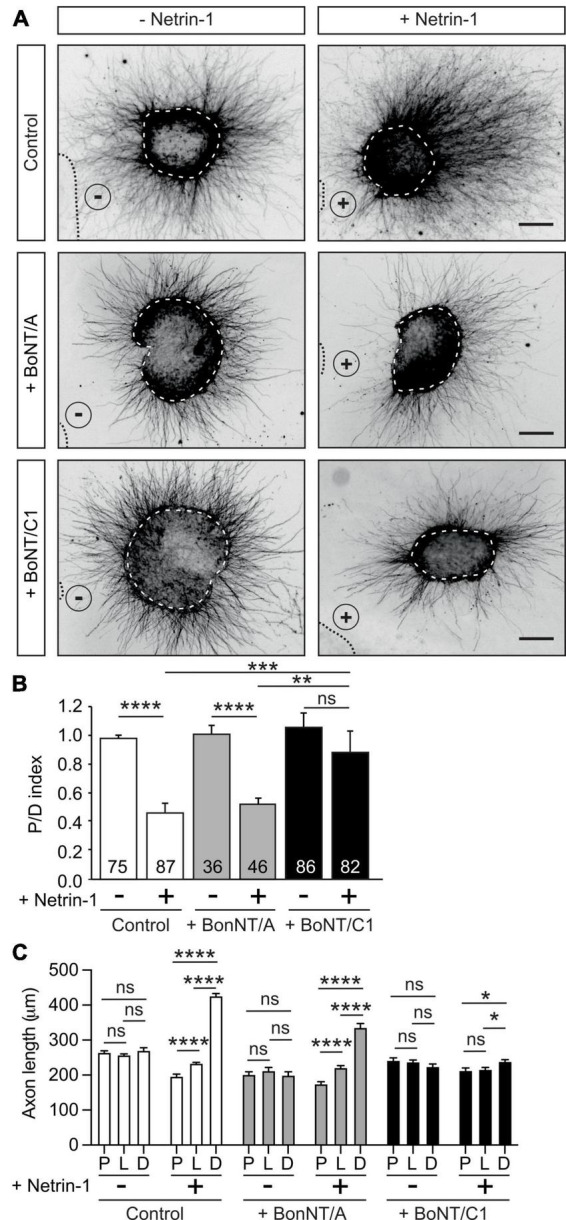
Cleavage of Stx1 by BoNT/C1 blocks Netrin-1-induced repulsion in EGL explants. **(A)** Representative images of EGL explants from P4 mice, immunodetected with anti-βIII-tubulin. Explants were confronted with control cell aggregates (−Netrin-1) or Netrin-1-secreting aggregates (+Netrin-1). Explants were cultured in the absence of BoNTs (control) or in medium supplemented with 25 nM BoNT/A (+BoNT/A) or 15 nM BoNT/C1 (BoNT/C1). HEK-293T aggregates are outlined with a dashed line. BoNT/C1, but not BoNT/A, cleaves Stx1 thereby abolishing the netrin-induced repulsion. Scale bar represents 100 μm. **(B)** Graph showing the calculated P/D ratio. **(C)** Graph showing the measured total axonal length. P stands for proximal explant quadrant; L stands for lateral explant quadrant; D stands for distal explant quadrant. Data represent mean ± SEM. One-way ANOVA followed by Dunn’s multiple comparison *post-hoc* test of selected pairs was used in panel **(B)**. One-way ANOVA followed by Games-Howel or by Dunet T3’s multiple comparison *post-hoc* test was used in panel **(C)**. The number of explants analyzed for each condition is indicated within each bar, ranging from 36 to 87 explants. 25 axons were averaged per explant quadrant. The results were generated from at least three independent experiments. ns stands for non-significant, *p ≤ 0.05, **p ≤ 0.01, ***p ≤ 0.001, ****p ≤ 0.0001.

Netrin-1 exerts long-range chemorepulsion that is important for axonal guidance (Round and Stein, 2007), and plays a major role in the organization of EGL neurons during early postnatal development of the cerebellum, controlling the growth of parallel fibers (Przyborski et al., 1998; Alcantara et al., 2000; Peng et al., 2010; Consalez et al., 2020). EGL neurons have been shown to co-express DCC, neogenin, DSCAM, UNC5B, and UNC5C that sense Netrin-1 expressed in the EGL and interneurons of the molecular layer of the cerebellum (Alcantara et al., 2000; Kim and Ackerman, 2011; Hernaiz-Llorens et al., 2020). To investigate whether Stx1 is necessary for Netrin-1-dependent repulsion, we co-cultured postnatal EGL explants in type I collagen 3D hydrogels and exposed them to aggregates of either control HEK-293T cells or Netrin-1-expressing HEK-293T cells for 2 days (Alcantara et al., 2000; Hernaiz-Llorens et al., 2020). EGL explants cultured with control HEK-293T cells exhibited a radial pattern of axonal growth (Figure 2A). In contrast, those confronted with Netrin-1-expressing cells showed strong chemorepulsion, with most axons growing in the distal quadrant (Figure 2A). Botulinum neurotoxins (BoNTs) are metalloproteases that reduce synaptic vesicle exocytosis and neurotransmitter release by cleaving specific SNARE proteins (Schiavo et al., 2000). Botulinum neurotoxin A (BoNT/A) exclusively cleaves SNAP25, whereas botulinum neurotoxin C1 (BoNT/C1) cleaves both Stx1 and SNAP25 (Blasi et al., 1993; Schiavo et al., 2000). Cleavage of Stx1 by BoNT/C1 in EGL cultures was confirmed by western blot (Supplementary Figure 3A). It has been shown that BoNTs cleave SNARE proteins in neuronal explants without affecting the secretion of Netrin-1 from stably transfected HEK-293T cells (Cotrufo et al., 2011). EGL-derived explants co-cultured with Netrin-1 in the presence of BoNT/C1 or BoNT/A displayed different phenotypes. While Netrin-1 elicited chemorepulsion in EGL explants incubated with BoNT/A, treatment with BoNT/C1 resulted in radial axonal sprouting (Figures 2A–C). Quantification of the length of axons sprouting from the explants confirmed a specific decrease in the length of explants treated with BoNT/A or BoNT/C1, but showed that only the use of BoNT/C1 toxin affected the chemorepulsive response toward Netrin-1 (Figure 2C).

As Stx1 interacts with UNC5 receptors but not with Neuropilin or Plexin receptors, we next investigated whether BoNTs altered Semaphorin 3A/3F-induced chemorepulsion (Supplementary Figure 3B). Hippocampal explants exhibited strong repulsion when confronted with Semaphorin 3A- or Semaphorin 3F-expressing cells. This repulsion was not altered when the explants were cultured in the presence of BoNT/A or BoNT/C1 (Supplementary Figure 3B). Taken together, these data indicate that the cleavage of Stx1 specifically abolishes Netrin-1-induced repulsion, but not class III Semaphorin-induced chemorepulsion.

### Postnatal Stx1B knock-out mice display decreased Netrin-1 chemorepulsion

**FIGURE 3:**
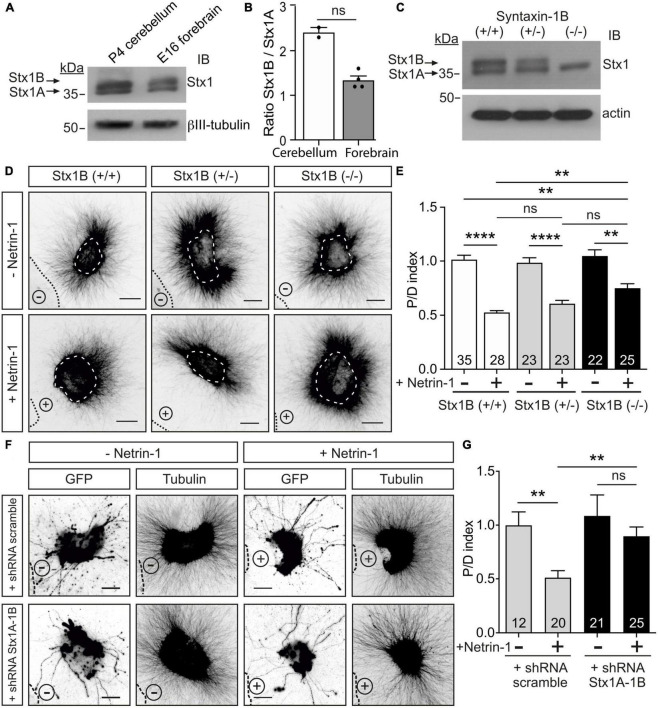
Downregulation of Stx1 blocks Netrin-1-induced repulsion in EGL explants. **(A)** Postnatal cerebellum and embryonic forebrain homogenates were subjected to urea/SDS-PAGE, resolving two Stx1 bands corresponding to Stx1B (upper band) and Stx1A (lower band). **(B)** Quantification of the ratio of intensities of Stx1B and Stx1A. **(C)** Western blot of embryonic forebrain homogenates from wild-type Stx1B (+/+), heterozygous Stx1B (+/−) and knock-out Stx1B (−/−) mice. The protein samples were subjected to urea/SDS-PAGE, resolving two Stx1 bands corresponding to Stx1B (upper band) and Stx1A (lower band). **(D)** Representative images of EGL explants from P4 mice of different genetic backgrounds for Stx1B: wild-type Stx1B (+/+), heterozygous Stx1B (+/−), knock-out Stx1B (−/−). Explants were immunodetected with anti-βIII-tubulin. Explants are outlined with a white dashed line. **(E)** Graph showing the calculated P/D ratio. **(F)** Representative images from EGL explants from P4 mice. Explants were immunodetected with anti-GFP and anti-βIII-tubulin (inset images). Explants were electroporated with a shRNA scrambled control plasmid, or with a shRNA plasmid against both Stx1A and Stx1B (shRNA Stx1A-1B). **(G)** Graph showing the calculated P/D ratio. Explants in panels **(D,F)** were confronted with control HEK-293T cell aggregates (−Netrin-1) or Netrin-1-secreting aggregates (+Netrin-1). Cell aggregates are outlined with a black dashed line in panels **(D,F)**. Scale bars in panels **(D,F)** represent 100 μm. Data in the plots in panels **(B,E,G)** represent mean ± SEM. Non-parametric Mann-Whitney test was performed in panel **(B)**, obtaining *p* = 0.133. One-way ANOVA followed by Dunn’s multiple comparison *post-hoc* test was used in panels **(E,G)**. Each data point plotted in panel **(B)** represents the western blot of one brain. The number of explants analyzed is indicated within each bar, ranging from 12 to 35 explants. Results were generated from at least three independent experiments. ns stands for non-significant, **p ≤ 0.01, ****p ≤ 0.0001.

Stx1 consists of two similar paralogs in mammals, Stx1A and Stx1B (Gerber et al., 2008; Ros et al., 2018). We investigated the expression of Stx1A and Stx1B in P3-5 postnatal cerebellum by western blot. We found that, although both paralogs are expressed, the expression pattern of the paralogs differs in the postnatal cerebellum and the forebrain (Figures 3A, B). It has previously been shown that mice deficient for Stx1A do not display anatomical abnormalities and only exhibit minor physiological neurotransmitter release deficits, most likely due to functional redundancy with Stx1B (Fujiwara et al., 2006). To evaluate the importance of Stx1B in Netrin-1-dependent chemorepulsion, we used neurons isolated from Stx1B knock-out mice (Ros et al., 2018). The level of Stx1B paralog was depleted in the knock-out mice (−/−), compared with the wild-type (+/+) or the heterozygous (±) animals (Figure 3C). Similar to what has already been described in other studies using independently generated Stx1B knock-out mice, newborn homozygous Stx1B (−/−) mice were slightly smaller than their control wild-type littermates (Supplementary Figure 4A; Kofuji et al., 2014; Wu et al., 2015). Whereas homozygous Stx1B knock-out mice usually survived until P7–P15, heterozygous Stx1B (±) targeted mice were viable until adulthood. We next performed Netrin-1 repulsion assays in EGL explants derived from P5 mice. EGL explants from control Stx1B (+/+) littermates displayed strong axonal chemorepulsion when confronted with Netrin-1-expressing cells. In contrast, chemorepulsion to Netrin-1 was significantly reduced in explants from homozygous Stx1B-deficient mice (Figures 3D, E) but was not completely abolished. To ascertain whether a functional redundancy with Stx1A may compensate for the lack of Stx1B, we generated double Stx1B/Stx1A-deficient mice. However, as double Stx1B/Stx1A knock-out animals die at birth (Ros et al., 2018), we used an alternative approach to target both Stx1 paralogs. We created a Stx1-shRNA sequence complementary to a conserved region in both Stx1A and 1B, enabling us to knock down both Stx1 paralogs. The efficiency of Stx1 downregulation was confirmed in PC12 cells, in which expression of the shRNA Stx1A-1B construct decreased Stx1 protein levels detected by western blot and made Stx1 undetectable by immunocytochemistry (Supplementary Figures 4B, C). Similar results were found after expressing the shRNA Stx1A-1B construct in EGL neurons and observing the decrease in Stx1 level (Supplementary Figure 4D). After electroporating EGL explants with the shRNA Stx1A-1B, the downregulation of both Stx1 paralogs resulted in a dramatic decrease in chemorepulsion, when compared to control cultures (Figures 3F, G). Together with the above results, these observations suggest that both Stx1 paralogs participate in Netrin-1-mediated chemorepulsion of EGL axons.

### Netrin-1 induces growth cone collapse associated with membrane internalization

**FIGURE 4:**
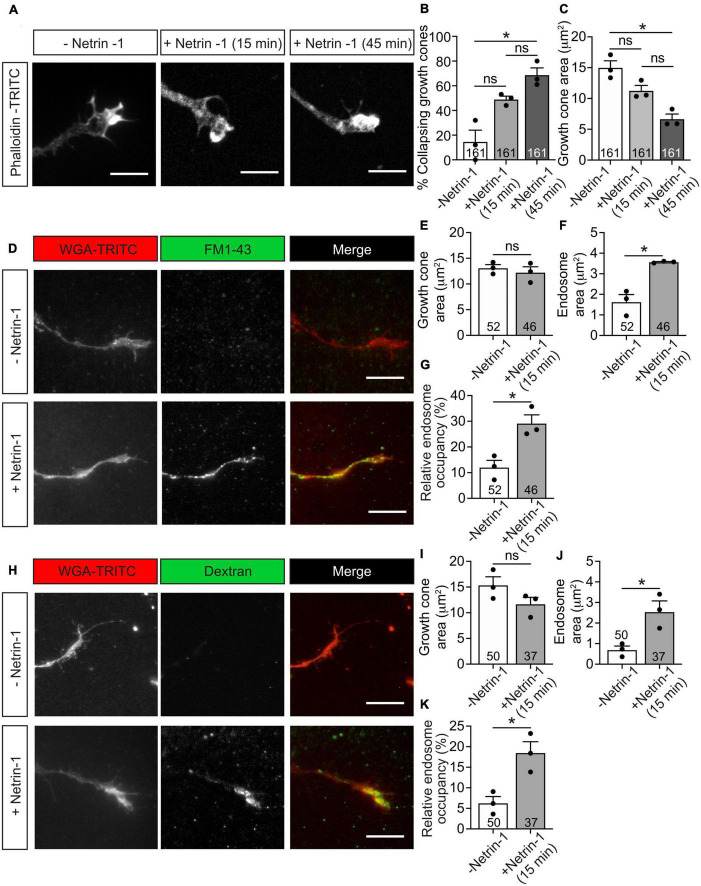
Netrin-1-induced growth cone collapse is associated with membrane internalization in EGL neurons. **(A)** Representative confocal images of growth cones from EGL neurons treated with either control medium or Netrin-1-supplemented medium (300 ng/mL, 15 or 45 min). Neurons were then immunostained with phalloidin-TRITC to detect actin cytoskeleton. Scale bar represents 10 μm. **(B)** The percentage of collapsing growth cones was calculated and plotted for each time point. **(C)**. Growth cone area was measured and plotted for each time point. **(D)** Representative confocal images of growth cones from EGL neurons treated with either control medium or Netrin-1-supplemented medium (300 ng/mL), together with FM1-43, for 15 min. Neurons were then labeled with the plasma membrane marker WGA-TRITC. Scale bar represents 10 μm. **(E)** Growth cone area was measured and plotted for each condition. **(F)** The total area occupied by vesicles within the growth cones was measured and plotted for each condition. **(G)** The percentage of the growth cone occupied by endocytic vesicles was calculated and plotted for each condition. **(H)** Representative confocal images of growth cones from EGL neurons treated with either control medium or Netrin-1-supplemented medium (300 ng/mL), together with Alexa Fluor 488-LMW-dextran, for 15 min. Neurons were then labeled with the plasma membrane marker WGA-TRITC. Scale bar represents 10 μm. **(I)** Growth cone area was measured and plotted in a graph for each condition. **(J)** The total area occupied by vesicles within the growth cones was measured and plotted for each condition. **(K)** The percentage of the growth cone occupied by endocytic vesicles was calculated and plotted for each condition. Data in panels **(B,C)** represent mean ± SEM. One-way ANOVA followed by Dunn’s multiple comparison *post-hoc* test was used. Each data point plotted represents one independent experiment (3 independent experiments in total per condition). A total of 51 to 55 growth cones were analyzed per condition. Data in panels **(D–F,H–J)** represent mean ± SEM. Unpaired two-tailed Student’s *t*-tests were used. Each data point plotted represents one independent experiment (3 independent experiments in total per condition). The number of growth cones analyzed is indicated within each bar; 11 to 20 growth cones were analyzed per experiment. ns stands for non-significant, *p ≤ 0.05.

To better understand the mechanisms involved in Netrin-1 chemorepulsion, we performed collapse assays in postnatal cerebellar EGL neurons. Primary neuronal cultures were exposed to Netrin-1 for 15–45 min and stained with phalloidin to label F-actin and analyze growth cone morphologies. Control cultures exhibited typical growth cones with triangular shapes and numerous lamellipodia and filopodia (Figure 4A). Incubation with Netrin-1 resulted in the initiation of the collapse of up to 75% of growth cones (Figures 4B, C), which exhibited the typical shriveled, round-type, pencil-like shape, largely devoid of filopodia or lamellipodia, with a redistribution of actin filaments toward the central domain of growth cones (Figure 4A).

Previous studies have shown that Semaphorin 3A (Fournier et al., 2000; Tojima et al., 2010) and Slit2 (Piper et al., 2006) induce endocytic events in growth cones. We therefore investigated whether Netrin-1 leads to endocytosis in EGL growth cones, using two typical markers of endocytosis, the styryl dye FM1-43 (Gaffield and Betz, 2007) and a fluorescently labeled low-molecular-weight (LMW, <10 kDa) dextran (Alexa Fluor 488-LMW-dextran) (Fournier et al., 2000). After incubation with Netrin-1 and the above dyes, EGL cultures were incubated with wheat germ agglutinin conjugated with TRITC (WGA-TRITC) to label the cell surface of growth cones (Figures 4D, H). After 15 min of Netrin-1 treatment, the growth cone areas were slightly reduced, ranging from 7 to 24% reduction (Figures 4C, E, I). The total area of internalized membranes (endosome area) within growth cones was also used to quantify the resulting endocytosis during growth cone collapse. Both markers used to monitor endocytosis showed that Netrin-1 triggers a marked increase in endosome area per growth cone (Figures 4F, J), as well as an increase in the percentage of the growth cone area occupied by internalized structures (endosome occupancy) (Figures 4G, K), showing that membrane internalization is an indicative parameter to appreciate changes during the initial phase of growth cone collapse, suggesting that the growth cone starts collapsing through the internalization of its cell surface components.

### Macropinocytosis, but not clathrin-dependent endocytosis, mediates Netrin-1-induced growth cone collapse and axon chemorepulsion

**FIGURE 5:**
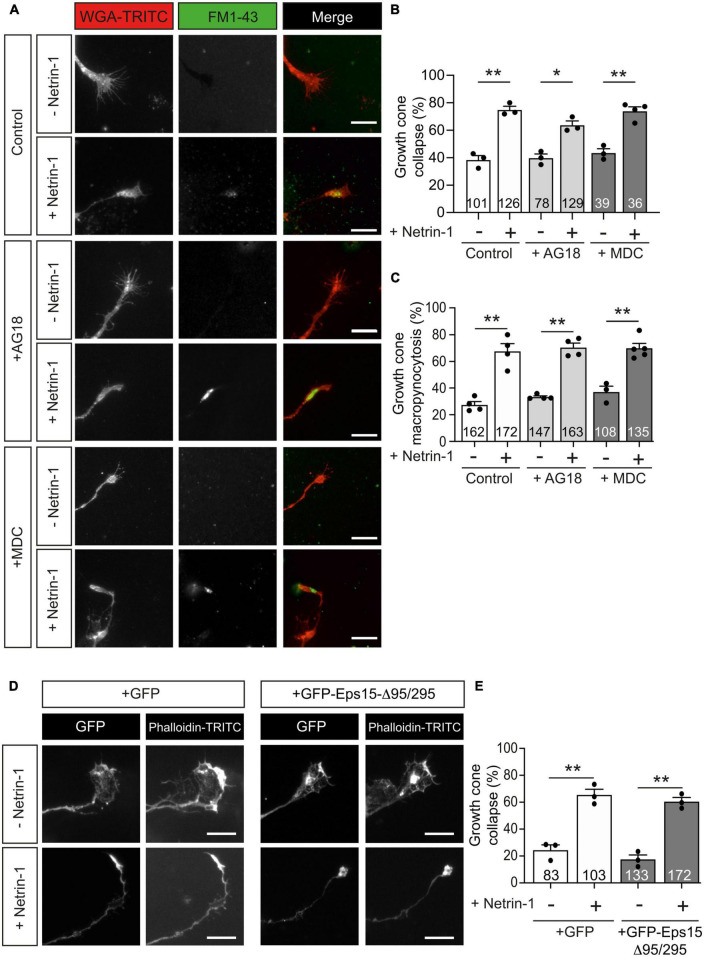
Netrin-1-induced growth cone collapse is independent of clathrin-dependent endocytosis in EGL neurons. **(A)** Representative confocal images of growth cones from EGL neurons treated with either control medium or Netrin-1-supplemented medium (300 ng/mL), together with FM1-43, for 45 min. A total of 30 min before the addition of control medium or Netrin-1, neurons were supplemented with control (DMSO) or with the clathrin-dependent internalization inhibitors MDC (1 μM), or AG18 (100 μM). These reagents were maintained during the subsequent 45 min incubation with Netrin-1. Neurons were then labeled with the plasma membrane marker WGA-TRITC. Scale bar represents 10 μm. **(B)** The percentage of collapsing growth cones was calculated and plotted for each condition. **(C)** The percentage of growth cones with internalized vesicles was plotted for each treatment. **(D)** Representative confocal images of growth cones from EGL neurons transfected with GFP or with the dominant negative GFP-Eps15-Δ95/295, and treated with either control medium or Netrin-1-supplemented medium (300 ng/mL, 45 min). Neurons were then immunostained with phalloidin-TRITC to detect actin cytoskeleton. Scale bar represents 10 μm. **(E)** The percentage of collapsing growth cones was plotted for each condition. Data plotted in panels **(B,C,E)** represent mean ± SEM. One-way ANOVA followed by Dunnett’s multiple comparison *post-hoc* test. Each data point represents one independent experiment. The number of growth cones analyzed is indicated within each bar From 27 to 129 growth cones were analyzed per experiment. *p ≤ 0.05, **p ≤ 0.01.

To test whether Netrin-1-induced growth cone collapse requires clathrin-dependent endocytosis, we used a combination of pharmacological and dominant negative strategies. Monodansylcadaverine (MDC) is a potent inhibitor of clathrin-dependent endocytosis (Schutze et al., 1999), and Tyrphostin A23 (AG18) is an inhibitor of the binding of internalized cargo to AP-2 (Banbury et al., 2003). We found that neither AG18 nor MDC was able to prevent the collapse of growth cones (Figures 5A, B) or the formation of Netrin-1-induced internalization of vesicles in growth cones (Figure 5C). Eps15-Δ95/295 is a dominant-negative form of Eps15 (EGFR pathway substrate clone 15) lacking the Eps15 homology domains. This truncated molecule inhibits the plasma membrane targeting of AP-2 and clathrin and the formation of coated pits and clathrin-dependent endocytosis (Benmerah et al., 1999; Poupon et al., 2002). Mutant Eps15-Δ95/295 overexpression in EGL neurons did not prevent growth cone collapse in neurons treated with Netrin-1 (Figures 5D, E). These results suggested that Netrin-1-induced large vesicle endocytosis and growth cone collapse are independent of clathrin-dependent internalization mechanisms.

**FIGURE 6:**
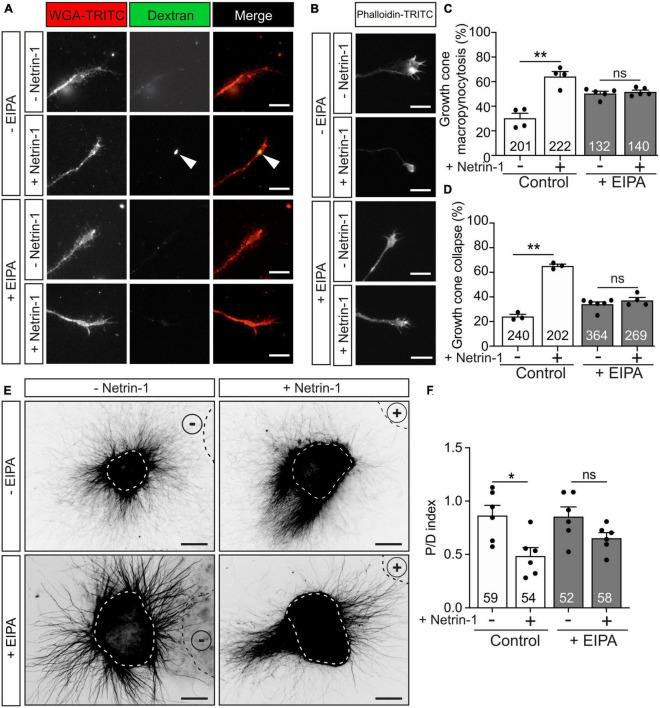
Netrin-1-induced growth cone collapse is dependent of macropinocytosis in EGL neurons. **(A)** Representative confocal images of growth cones from EGL neurons treated with either control medium or Netrin-1-supplemented medium (300 ng/mL), together with Fluorescein-HMW-dextran, for 45 min. A total of 10 min before the addition of control medium or Netrin-1, neurons were supplemented with control (DMSO) or with the macropinocytosis-specific inhibitor EIPA (10 μM). They were maintained on these supplements for the subsequent 15 min incubation with Netrin-1. Neurons were then labeled with the plasma membrane marker WGA-TRITC. Scale bar represents 10 μm. The arrowheads indicate dextran-positive macropinocytic structures. **(B)** Representative confocal images of growth cones from EGL neurons treated with either control medium or Netrin-1-supplemented medium (300 ng/mL) for 45 min. A total of 10 min before the addition of control medium or Netrin-1, neurons were supplemented with control (DMSO) or with the macropinocytosis-specific inhibitor EIPA (10 μM), which was maintained during the subsequent 45 min incubation with Netrin-1. Neurons were then immunostained with phalloidin-TRITC to detect actin cytoskeleton. Scale bar represents 10 μm. **(C)** The percentage of growth cones with macropinocytic vesicles plotted for each treatment. **(D)** The percentage of collapsing growth cones was plotted for each treatment. **(E)** Representative images of EGL explants from P4 mice, immunodetected with anti-βIII-tubulin. Explants are outlined with a dashed white line Explants were confronted with control cell aggregates (−Netrin-1) or Netrin-1-secreting aggregates (+Netrin-1). Explants were cultured in the absence of EIPA (control with DMSO) or in medium supplemented with 10 μM EIPA. HEK-293T aggregates are outlined with a dashed black line. Scale bar represents 100 μm. **(F)** Graph showing the calculated P/D ratio. Data in the plots in panels **(C,D,F)** represent mean ± SEM. One-way ANOVA followed by Dunn’s multiple comparison *post-hoc* test. The number of explants and growth cones analyzed is indicated within each bar. A total of 55 to 185 growth cones were analyzed per experiment. ns stands for non-significant, *p ≤ 0.05, **p ≤ 0.01.

Macropinocytosis is a clathrin-or-caveolin-independent form of endocytosis that forms large endocytic vacuoles (Ferreira and Boucrot, 2018; Lin et al., 2020). The incorporation of high-molecular-weight (HMW, >10 kDa) dextran has been associated with membrane retrieval by macroendocytic vesicles (Kabayama et al., 2009; Kolpak et al., 2009). We investigated Netrin-1-induced membrane retrieval through macropinocytosis by analyzing the internalization of Fluorescein-HMW-dextran in the absence or presence of 5-(N-ethyl-N-isopropyl) amiloride (EIPA), a potent analog of amiloride channels that has been used as a macropinocytosis-specific inhibitor (Meier et al., 2002; Koivusalo et al., 2010; Sundaramoorthy et al., 2013; Tian et al., 2014; Zeineddine and Yerbury, 2015; Costa Verdera et al., 2017). Our results revealed that incubation with Netrin-1 induced the internalization of HMW-dextran (Figure 6A, arrowhead). Pre-incubation with EIPA resulted in a minor increase in basal macropinocytosis, but the Netrin-1-dependent macropinocytosis was entirely blocked by pre-treatment with EIPA (Figures 6A, C). We next asked whether the blockade of macropinocytosis also influenced Netrin-1-induced growth cone collapse. Incubation with EIPA was also able to dramatically abolish Netrin-1-induced growth cone collapse in EGL growth cones (Figures 6B, D). To evaluate whether axonal chemorepulsion to Netrin-1 was also associated with macropinocytosis, postnatal EGL explants were grown confronted to Netrin-1 in the presence of EIPA. Our results showed that the blockade of macropinocytosis with EIPA could inhibit the axonal chemorepulsion toward Netrin-1 (Figures 6E, F). Taken together, our data indicate that macropinocytosis, but not clathrin-dependent endocytosis, mediates Netrin-1-induced growth cone collapse and axon repulsion in EGL axons.

### Stx1 blockade inhibits Netrin-1-induced growth cone collapse and macropinocytosis

**FIGURE 7:**
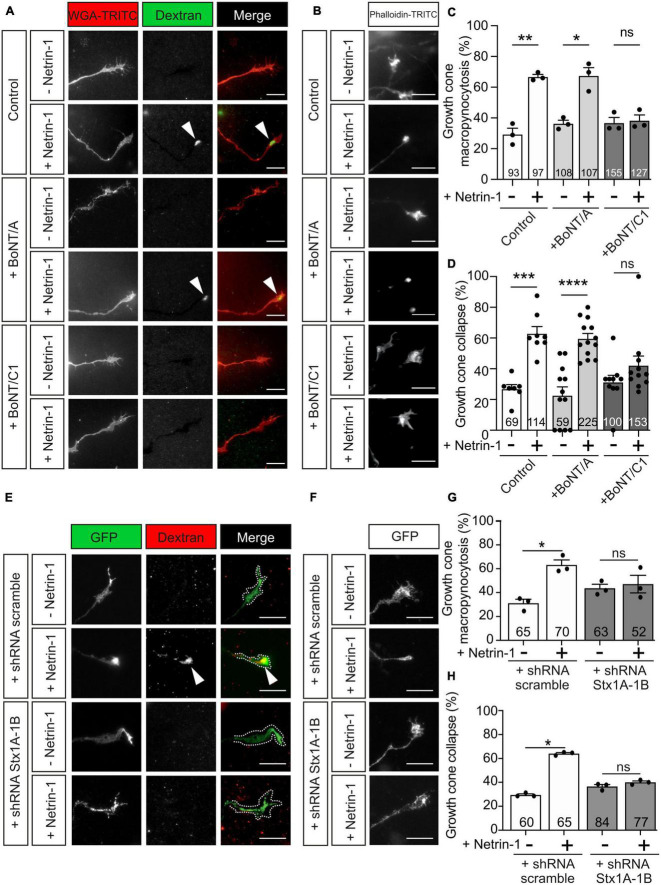
Netrin-1-induced growth cone collapse requires Stx1 in EGL neurons. **(A)** Representative confocal images of growth cones from EGL neurons treated with either control medium or Netrin-1-supplemented medium (300 ng/mL), together with Fluorescein-HMW-dextran, for 45 min. A total of 15 min before the addition of control medium or Netrin-1, neurons were supplemented with control (PBS) or 25 nM BoNT/A (+BoNT/A) or 15 nM BoNT/C1 (BoNT/C1). The toxins were maintained during the subsequent 15 min incubation with Netrin-1. Neurons were then labeled with the plasma membrane marker WGA-TRITC. Scale bar represents 10 μm. The arrowheads indicate dextran-positive macropinocytic structures. **(B)** Representative confocal images of growth cones from EGL neurons treated with either control medium or Netrin-1-supplemented medium (300 ng/mL) for 45 min. A total of 15 min before the addition of control medium or Netrin-1, neurons were supplemented with control (PBS) or 25 nM BoNT/A (+BoNT/A) or 15 nM BoNT/C1 (BoNT/C1). The toxins were maintained during the subsequent 45 min incubation with Netrin-1. Neurons were then immunostained with phalloidin-TRITC to detect actin cytoskeleton. Scale bar represents 10 μm. **(C)** The percentage of growth cones with macropinocytic vesicles was calculated and plotted in a graph for each treatment. **(D)** The percentage of collapsing growth cones was plotted for each treatment. **(E)** Representative confocal images of growth cones from EGL neurons transfected with a shRNA scrambled control plasmid, or with a shRNA plasmid against both Stx1A and Stx1B (shRNA Stx1A-1B), and treated with either control medium or Netrin-1-supplemented medium (300 ng/mL), together with tetramethylrhodamine-HMW-dextran, for 45 min. Neurons were then immunostained anti-GFP to detect transfected growth cones. Scale bar represents 10 μm. The arrowhead indicates dextran-positive macropinocytic structures. **(F)** Representative confocal images of growth cones from EGL neurons transfected with a shRNA scrambled control plasmid, or with a shRNA plasmid against both Stx1A and Stx1B (shRNA Stx1A-1B), and treated with either control medium or Netrin-1-supplemented medium (300 ng/mL), for 45 min. Neurons were then immunostained with anti-GFP to detect transfected growth cones. Scale bar represents 10 μm. **(G)** The percentage of growth cones with macropinocytic vesicles was plotted for each treatment. **(H)** The percentage of collapsing growth cones was plotted for each treatment. Data of the plots in panels **(C,D,G,H)** represent mean ± SEM. One-way ANOVA followed by Sidak’s multiple comparison *post-hoc* test of selected pairs was used in panels **(C,D)**. One-way ANOVA followed by Dunn’s multiple comparison *post-hoc* test was performed in panels **(G,H)**. The number of explants analyzed is indicated within each bar. A total of 7 to 68 growth cones were analyzed per experiment. ns stands for non-significant, *p ≤ 0.05, **p ≤ 0.01, ***p ≤ 0.001, ****p ≤ 0.0001.

It has recently been reported that Stx1 is involved in rapid and slow endocytosis at synapses, which can be either clathrin-independent or -dependent (Xu et al., 2013). As Stx1 interacts with UNC5 receptors, which mediate chemorepulsion and growth cone collapse, we next determined whether Netrin-1-induced growth cone macropinocytosis and collapse were Stx1-dependent using BoNTs. BoNT/A or BoNT/C1 was added to the medium 10 min before incubation with Netrin-1. The percentage of macropinocytic vesicle-containing growth cones increased about twofold when Netrin-1 was added alone or in the presence of BoNT/A (Figures 7A, C). In contrast, Netrin-1-induced formation of macropinocytic vesicles was reduced specifically by cleaving Stx1 using BoNT/C1 (Figures 7A, C). We then tested whether BoNT/A or BoNT/C1 altered growth cone collapse. Neurons treated with control medium or with control medium supplemented with BoNT/A or BoNT/C1 exhibited normal growth cone morphologies with lamellipodia and filopodia (Figure 7B). After incubation with Netrin-1, about 70% of EGL growth cones exhibited collapsing and round-tipped shapes (Figures 7B, D). This effect was maintained when EGL neurons were co-incubated with BoNT/A, but was markedly reduced when the cultures were co-incubated with BoNT/C1 (Figure 7D). These results suggested that Stx1 is necessary for both Netrin-1-induced growth cone collapse and macropinocytosis.

To confirm these findings, we downregulated both Stx1 paralogs using the Stx1A-1B shRNA construct. Electroporation of EGL cells with Stx1A-1B shRNA, but not with control scrambled shRNA sequence, blocked the formation of macropinocytic vesicles in EGL growth cones incubated with Netrin-1 (Figures 7E, G). Similarly, the knockdown of Stx1 blocked Netrin-1-dependent collapsing of growth cones (Figures 7F, H). Our results, therefore, demonstrate that Stx1 is required for both Netrin-1-dependent growth cone collapse and macropinocytosis.

## Discussion

The dynamics of growth cones are essential for axonal guidance and the establishment of neuronal connectivity. How the growth cone transduces guidance cue signaling into cellular responses has received notable attention. The motile nature of the growth cone requires fine control of cytoskeletal and membrane dynamics (Blanquie and Bradke, 2018; McCormick and Gupton, 2020). The growth cone is known to be a major site of endo- and exocytosis. However, the mechanisms that regulate these processes in response to chemoattractive or chemorepulsive cues are still being unveiled. Based on the results of the current study, we propose a new mechanism involving the close interplay between signaling receptors and membrane homeostasis using the SNARE protein Stx1. We show a biochemical interaction between the repulsive Netrin-1 receptor UNC5 and the SNARE protein Stx1. We also demonstrate that macropinocytosis, and not clathrin-dependent endocytosis, is the membrane retrieval process mediating repulsive responses to Netrin-1. The absence of Stx1 or interference with its function blocks repellent Netrin-1 responses in EGL cells, from membrane endocytosis to chemorepulsion.

Two main mechanisms of membrane retrieval have been associated with axon guidance: clathrin-dependent endocytosis and macropinocytosis (Kabayama et al., 2009; Kolpak et al., 2009; Tojima et al., 2010). We investigated growth cone endocytosis through two complementary approaches, staining newly formed vesicles with FM dyes and with fluorescent dextrans. FM dyes have previously been used to study growth cone endocytosis, staining a wide plethora of vesicular structures derived from the plasmalemma, with different morphologies and sizes (Hines et al., 2010). The incorporation of HMW dextrans into large vesicles has been associated with macroendocytic events (Swanson and Watts, 1995; Kabayama et al., 2009; Kolpak et al., 2009). When combined, HMW dextran-positive vesicles account for less than half of those stained with FM dyes, indicating the selectivity of HMW toward macroendocytosis-derived vesicles (Clayton and Cousin, 2009). Our data reproduce this phenomenon and are consistent with the existence of at least two vesicle pools regulated by distinct signaling cascades, one of them being large vesicles generated by macropinocytosis derived from the events associated with the repulsion toward Netrin-1. Recent studies have described the mechanisms underlying endocytic processes in response to class III semaphorins, but the characterization of membrane dynamics during repulsive responses to Netrin-1 has yet to be addressed (Fournier et al., 2000; Jurney et al., 2002; Itofusa et al., 2017; Boyer and Gupton, 2018). In cortical growth cones, the repulsive actions of Semaphorin-3A have been hypothesized to require a VAMP-2-dependent mechanism of receptor sorting followed by clathrin-dependent endocytosis of the local growth cone membrane (Zylbersztejn et al., 2012). Our results suggest that the Netrin-1-dependent EGL growth cone collapse and retraction are not affected by the inhibition of clathrin-dependent coated pit formation of endocytic vesicles. On the other hand, macropinocytosis is considered the main cellular mechanism to uptake large volumes of liquid and membrane. Macropinosomes can be generated by actin-dependent membrane ruffling or by the fusion of multiple membrane ruffles with the plasma membrane, allowing the incorporation of HMW molecules such as dextrans (Swanson and Watts, 1995). Our data show an association between Netrin-1 treatment and EGL growth cone collapse followed by the formation of large, HMW dextran-positive vesicles. Not only the increase of dextran incorporation was abolished in the presence of the macropinocytosis-specific inhibitor EIPA (Meier et al., 2002; Kabayama et al., 2009; Koivusalo et al., 2010; Sundaramoorthy et al., 2013; Tian et al., 2014; Zeineddine and Yerbury, 2015; Costa Verdera et al., 2017), but also the suppression of Netrin-1-induced growth cone collapse and the retraction of EGL axons. These findings demonstrate for the first time that Netrin-1-induced chemorepulsion relies on macropinocytosis as the mechanism for growth cone membrane retrieval, in contrast to the repulsion to class-III semaphorins (Zylbersztejn et al., 2012), and analogous to Shh repulsion (Kolpak et al., 2009). Our study, therefore, confirms that repulsive guidance relies on multiple membrane retrieval mechanisms whereby a given set of growth-inhibiting molecules associate with different signaling pathways to drive endocytosis.

Netrin-1 was the first guidance cue to be described, but the intricacies of its functions are still being revealed (Boyer and Gupton, 2018). Netrin-1 exerts a strong role during cerebellar development, controlling granule cell migration, and regulating the formation of contralateral projections toward the midline, the formation of ipsilateral projections during the extension of parallel fibers, and the development of olivo-cerebellar projections (Bloch-Gallego et al., 1999). Downregulation of proteins involved in the downstream signaling cascades associated with growth cone repulsion is associated with the alteration of granule cell migration from the EGL and unresponsiveness to Netrin-1 (Peng et al., 2010). These results suggest a predominant role of Netrin-1 in correct cerebellar formation. Our results support these studies and offer a new view of this mechanism, whereby the induction of macropinocytosis underlines the repulsive effects of Netrin-1.

SNARE proteins are expressed in the developing brain and axons, and their role beyond synaptic transmission during axon navigation is just being unveiled. VAMP-2 function is required for repulsion to class III semaphorins (Zylbersztejn et al., 2012), and Stx1A is involved in Slit/Robo midline axonal repulsion (Ros et al., 2018). We have previously shown how Stx1 mediates ligand-dependent exocytosis and attraction to neurotrophic factors (Fuschini et al., 2018) and Netrin-1 (Cotrufo et al., 2011, 2012) through the interaction with Trk receptors and DCC, respectively. Here we describe how Stx1 interacts directly with the repulsive Netrin-1 receptor UNC5. The repulsive guidance of EGL axons was severely impaired when interfering with Stx1 function through (i) its cleaving with BoNT/C1, (ii) its suppression in knock-out animals, or (iii) its downregulation using specific shRNAs. Both Stx1A and 1B are abundantly expressed in the brain (Aguado et al., 1999) and share basic functions as neuronal t-SNAREs. However, only Stx1B is necessary for the regulation of spontaneous and evoked synaptic vesicle exocytosis in fast transmission (Mishima et al., 2014), which suggests that compensatory mechanisms underlie the mild impairment in basal synaptic transmission reported in Stx1A knock-out animals (Fujiwara et al., 2006). In addition, Stx1B is essential for spontaneous GABAergic transmission frequency in the cerebellum, most likely attributed to a lower number of neurons, suggesting differential importance of Stx1B for neuronal survival (Park et al., 2014; Wu et al., 2015). Our approach using Stx1B knock-out mice only partially reduced the repulsive effects of Netrin-1 on EGL neurons. Experiments with Stx1B (+/+), (+/−), and (−/−) animals showed an incremental dose dependence in the severity of their phenotypes, appointing to a partial functional compensation. Additional experiments in double knock-out animals may have confirmed this compensatory effect, but the embryonic lethality of these mice precluded such studies.

Growth cone receptors transduce their signaling during axon guidance through complex mechanisms that can be influenced by several factors, including the effective amount of receptors located on the surface of growth cones, their dynamic membrane properties, and their signaling mechanisms. At any given time, in addition to the receptors anchored in the plasmalemma, there are also intracellular receptors in endocytosis or micropinocytosis vesicles, in sorting organelles, and in *en route* intracellular vesicles originating from the anterograde biosynthetic pathway (O’Donnell et al., 2009). Regulating the surface levels of axon guidance receptors represents an important mechanism to control the response of growing axons toward extracellular cues. For example, DCC is exocytosed in the presence of Netrin-1 in a process enabling the interaction with Stx1 (Matsumoto and Nagashima, 2010; Cotrufo et al., 2011). The organization of receptors in membrane microdomains can also modulate their activity. UNC5 distribution in lipid raft cholesterol-enriched microdomains is crucial to its growth cone repulsive response to Netrin-1 in postnatal cerebellar EGL neurons (Hernaiz-Llorens et al., 2020). Specific endocytosis of transmembrane receptors on the surface of growth cones can diminish and even reverse axon responses to extracellular signals by controlling the total amount of available receptors. Protein kinase C (PKC) activation initiates the formation of a protein complex between the protein interacting with C-kinase 1 (Pick1), PKC and UNC5H1 that will lead to selective internalization of UNC5H1 from the surface of growth cones, a concomitant increased colocalization with early endosomal markers, and a reduced Netrin-1-chemorepulsion (Williams et al., 2003; Bartoe et al., 2006; O’Donnell et al., 2009). PKC has been linked to macropinocytosis in macrophages (Singla et al., 2019). However, no evidence has linked PKC activation with Netrin-1 signaling during axon guidance, and only a Netrin-1-independent adenosine A2b receptor (A2bR) signaling has been shown to initiate UNC5A internalization through PKCα activation (McKenna et al., 2008). These pieces of evidence suggest that PKC-dependent regulation of UNC5-Netrin-1 chemorepulsion is not likely to happen in EGL neurons during postnatal cerebellar development. Our findings indicate that Netrin-1 triggers the internalization of large amounts of extracellular membrane through macropinocytosis, resulting in growth cone collapse. Whether this leads to a UNC5/DCC surface imbalance that favors a Netrin-1-dependent axon regrowth will require further investigation.

The type of signaling mechanism is very diverse among axon guidance receptors (Pasterkamp and Burk, 2021). UNC5 and DCC Netrin-1 receptors initiate their signaling on the surface of growth cones (Boyer and Gupton, 2018). However, numerous ligands such as neurotrophins (Ginty and Segal, 2002) or ephrin (O’Donnell et al., 2009) continue their signaling once their receptors have been internalized. Since Netrin-1 is an extracellular guiding molecule, we believe that the first activation of UNC5 occurs in the plasmalemma, although we cannot rule out that Netrin-1 receptors may continue signaling once internalized. Netrin-1 stimulation triggers DCC ubiquitination and internalization, leading to its degradation via the proteasome pathway (Kim et al., 2005). Trafficking routes of internalized UNC5, however, remain to be deciphered. Interestingly, retrograde NGF-TrkA and BDNF-TrkB neurotrophin signaling is first initiated by macropinocytic mechanisms in superior cervical ganglia (SCG) neurons, leading to the formation of retrograde signaling macroendosomes that sustain neuronal survival (Valdez et al., 2005). The interaction of internalized UNC5 receptors with possible downstream targets and whether these UNC5-containing endocytic vesicles remain active will be the object of future studies.

Our work demonstrates for the first time that Stx1 is required for Netrin-1-induced macropinocytosis and growth cone collapse. It has previously been shown that Stx1 and other SNARE proteins also play a role in endocytosis at synapses (Xu et al., 2013; Zhang et al., 2013), but no mechanistic explanation has been proposed. Our experiments using toxins further indicate that Stx1 function, and not synaptic fusion mediated by the SNARE complex, is required to transduce Netrin-1 chemorepulsion, given that BoNT/A treatments, which specifically disrupt SNAP-25 but do not affect Stx1, do not block repulsion to Netrin-1. In addition, they provide mechanistic insight, as interfering with Stx1 function using BoNT/C1 or shRNA also blocks the Netrin-1-induced increase in macropinocytosis that precedes growth cone collapse. Previous reports showed that Stx1 downregulation enhances basal growth cone collapse and macropinocytosis in DRG neurons (Igarashi et al., 1996; Kabayama et al., 2011). The authors suggested a potential imbalance in newly exocytosed membrane retrieval due to the absence of Stx1, combined with the activation of a Rac1-dependent macropinocytic pathway. This hypothesis does not completely explain the blockade of macropinocytosis and collapse upon Netrin-1 treatments that we observed. Our findings indicate that the absence and blocking of Stx1 have variable effects during basal growth cone macropinocytosis and axon extension, suggesting fundamental differences in the control of growth cone collapse during basal conditions and in the presence of different guidance cues. Whether these differences are associated with particular expression of specific SNARE proteins and guidance receptors, and their mutual interactions will require further investigations.

In previous reports, we showed that Stx1 forms a protein complex with the Netrin-1 receptor DCC, coupling the chemotropic Netrin-1/DCC axonal attraction and growth cone SNARE-dependent exocytic fusion of vesicles with extracellular membranes (Cotrufo et al., 2011). In agreement with these findings, here we demonstrated that the t-SNARE Stx1 can also interact physically with the Netrin-1 receptor UNC5, thereby mediating cerebellar repulsion. Unfortunately, the precise mechanism behind Stx1-UNC5 association and chemorepulsion remains unknown. Interestingly, both Stx1A and UNC5C receptors organize forming membrane nanoclusters (Bademosi et al., 2017; Hernaiz-Llorens et al., 2020). The association of UNC5 with Stx1 could facilitate the organization of UNC5 into functional nanoclusters, and alteration of these nanostructures can disrupt their function. Alternatively, Stx1 could also affect the exocytic delivery of new UNC5 receptors to the surface of EGL growth cones, thus affecting the resulting chemorepulsive response. This type of exocytic model has been postulated to account for Vamp2 action during Sema3A chemorepulsion (Zylbersztejn et al., 2012).

Binding studies have found that Stx1 interacts with Dynamin-2 during secretion in adrenal chromaffin cells (Galas et al., 2000). It has been reported that Dynamin-2 is involved in clathrin-dependent endocytosis (Bertot et al., 2018) and in clathrin-independent macropinocytosis induced by Shh (Kolpak et al., 2009). Thus, Dynamin-2 could mediate the macropinocytic response to Netrin-1 in cooperation with Stx1 and UNC5 receptors. A parallel role for Stx1 could be facilitating the formation of the pseudopodial extension that precedes phagosome formation, as it requires focal exocytosis and reorganization of the actin cytoskeleton (Lim and Gleeson, 2011). The mechanisms required for focal exocytosis in macrophage macropinocytosis resemble those involved in neuronal growth cone extension, as both require SNARE proteins such as Ti-VAMP (Braun et al., 2004; Cotrufo et al., 2011) to provide the new membrane that is necessary for extension. A net loss of actin has been described in collapsed growth cones, and F-actin bundles progressively disappear from retracting filopodia (Fan et al., 1993). In the initial stages of growth cone collapse, actin reorganizes from bundles to a meshwork structure, followed by a redistribution of actin filaments away from the leading edge toward the central domain (Zhou and Cohan, 2001). Our results support the idea that the internalization of cell surface components through macropinocytosis is an initial step during growth cone collapse, pointing to an active involvement of the actin cytoskeleton. Unfortunately, the precise mechanism that triggers membrane internalization remains unclear. It has recently been described that axonal retraction in dendrites of the nematode *C. elegans* depends on UNC-6 (Netrin)-UNC-40 (DCC)-UNC-5 interaction, which activates actin polymerization and facilitates retraction through the involvement of NMY-1 (non-muscle myosin II) (Sundararajan et al., 2019). Interestingly, non-muscle myosin IIA and B are involved in growth cone collapse and neurite retraction (Costa and Sousa, 2020). Additional experimentation to investigate the mechanistic machinery involving Stx1 during Netrin-1/UNC5-induced growth cone chemorepulsion is warranted.

In conclusion, attractive and repulsive responses to Netrin-1 use a parallel approach to handle membrane incorporation and retrieval. Both DCC and UNC5 rely on Stx1 to initiate membrane exocytosis or endocytosis and ultimately attractive or repulsive growth cone responses. This opens a new scenario whereby key proteins share multiple apparently opposite roles depending on the context of the signaling molecules present.

## Consent for publication

All the authors involved in the study have read it and provided their consent for publication.

## Data availability statement

The raw data supporting the conclusions of this article will be made available by the authors, without undue reservation.

## Ethics statement

The animal study was approved by the CEEA of the University of Barcelona. The study was conducted in accordance with the local legislation and institutional requirements. All the authors involved in the study have read it and provided their consent for publication.

## Author contributions

RM-M: Conceptualization, Data curation, Formal analysis, Investigation, Methodology, Supervision, Validation, Writing—original draft, Writing—review and editing. AM: Data curation, Methodology, Writing—review and editing. TC: Data curation, Investigation, Methodology. CR-B: Data curation, Investigation, Methodology. OR: Data curation, Investigation, Methodology, Writing—review and editing. MH-L: Data curation, Investigation, Methodology, FP-B: Data curation, Investigation, Methodology. RA: Data curation, Investigation, Methodology. AP: Investigation, Methodology, Writing—review and editing. MP: Data curation, Investigation, Methodology. FU: Data curation, Investigation, Methodology. ES: Conceptualization, Funding acquisition, Investigation, Resources, Supervision, Writing—original draft, Writing—review and editing.

## References

[B1] Aguado F. Majo G. Ruiz-Montasell B. Llorens J. Marsal J. Blasi J. (1999). Syntaxin 1A and 1B display distinct distribution patterns in the rat peripheral nervous system. *Neuroscience* 88 437–446. 10.1016/s0306-4522(98)00247-4 10197765

[B2] Alcantara S. Ruiz M. De Castro F. Soriano E. Sotelo C. (2000). Netrin 1 acts as an attractive or as a repulsive cue for distinct migrating neurons during the development of the cerebellar system. *Development* 127 1359–1372. 10704383 10.1242/dev.127.7.1359

[B3] Bademosi A. Lauwers E. Padmanabhan P. Odierna L. Chai Y. Papadopulos A. (2017). In vivo single-molecule imaging of syntaxin1A reveals polyphosphoinositide- and activity-dependent trapping in presynaptic nanoclusters. *Nat. Commun.* 8:13660. 10.1038/ncomms13660 PMC5171881 28045048

[B4] Banbury D. Oakley J. Sessions R. Banting G. (2003). Tyrphostin A23 inhibits internalization of the transferrin receptor by perturbing the interaction between tyrosine motifs and the medium chain subunit of the AP-2 adaptor complex. *J. Biol. Chem.* 278 12022–12028. 10.1074/jbc.M211966200 12556528

[B5] Barrecheguren P. Ros O. Cotrufo T. Kunz B. Soriano E. Ulloa F. (2017). SNARE proteins play a role in motor axon guidance in vertebrates and invertebrates. *Dev. Neurobiol.* 77 963–974. 10.1002/dneu.22481 28033683

[B6] Bartoe J. McKenna W. Quan T. Stafford B. Moore J. Xia J. (2006). Protein interacting with C-kinase 1/protein kinase Calpha-mediated endocytosis converts netrin-1-mediated repulsion to attraction. *J. Neurosci.* 26 3192–3205. 10.1523/JNEUROSCI.3469-05.2006 16554470 PMC6674106

[B7] Benmerah A. Bayrou M. Cerf-Bensussan N. Dautry-Varsat A. (1999). Inhibition of clathrin-coated pit assembly by an Eps15 mutant. *J. Cell Sci.* 112 1303–1311. 10194409 10.1242/jcs.112.9.1303

[B8] Bertot L. Grassart A. Lagache T. Nardi G. Basquin C. Olivo-Marin J. (2018). Quantitative and statistical study of the dynamics of clathrin-dependent and -independent endocytosis reveal a differential role of endophilin A2. *Cell Rep.* 22 1574–1588. 10.1016/j.celrep.2018.01.039 29425511

[B9] Blanquie O. Bradke F. (2018). Cytoskeleton dynamics in axon regeneration. *Curr. Opin. Neurobiol.* 51 60–69. 29544200 10.1016/j.conb.2018.02.024

[B10] Blasi J. Chapman E. Link E. Binz T. Yamasaki S. De Camilli P. (1993). Botulinum neurotoxin A selectively cleaves the synaptic protein SNAP-25. *Nature* 365 160–163. 8103915 10.1038/365160a0

[B11] Bloch-Gallego E. Ezan F. Tessier-Lavigne M. Sotelo C. (1999). Floor plate and netrin-1 are involved in the migration and survival of inferior olivary neurons. *J. Neurosci.* 19 4407–4420. 10341242 10.1523/JNEUROSCI.19-11-04407.1999 PMC6782586

[B12] Bouchard J. Horn K. Stroh T. Kennedy T. (2008). Depolarization recruits DCC to the plasma membrane of embryonic cortical neurons and enhances axon extension in response to netrin-1. *J. Neurochem.* 107 398–417. 10.1111/j.1471-4159.2008.05609.x 18691385

[B13] Bouchard J. Moore S. Tritsch N. Roux P. Shekarabi M. Barker P. (2004). Protein kinase A activation promotes plasma membrane insertion of DCC from an intracellular pool: A novel mechanism regulating commissural axon extension. *J. Neurosci.* 24 3040–3050. 10.1523/JNEUROSCI.4934-03.2004 15044543 PMC6729852

[B14] Boyer N. Gupton S. (2018). Revisiting netrin-1: One who guides (axons). *Front. Cell Neurosci.* 12:221. 10.3389/fncel.2018.00221 30108487 PMC6080411

[B15] Braun V. Fraisier V. Raposo G. Hurbain I. Sibarita J. Chavrier P. (2004). TI-VAMP/VAMP7 is required for optimal phagocytosis of opsonised particles in macrophages. *EMBO J.* 23 4166–4176. 10.1038/sj.emboj.7600427 15470500 PMC524391

[B16] Brunger A. Choi U. Lai Y. Leitz J. White K. Zhou Q. (2019). The pre-synaptic fusion machinery. *Curr. Opin. Struct. Biol.* 54 179–188. 30986753 10.1016/j.sbi.2019.03.007 PMC6939388

[B17] Clayton E. Cousin M. (2009). Quantitative monitoring of activity-dependent bulk endocytosis of synaptic vesicle membrane by fluorescent dextran imaging. *J. Neurosci. Methods* 185 76–81. 10.1016/j.jneumeth.2009.09.016 19766140 PMC3458301

[B18] Consalez G. Goldowitz D. Casoni F. Hawkes R. (2020). Origins, development, and compartmentation of the granule cells of the cerebellum. *Front. Neural Circ.* 14:611841. 10.3389/fncir.2020.611841 33519389 PMC7843939

[B19] Costa A. Sousa M. (2020). Non-muscle myosin II in axonal cell biology: From the growth cone to the axon initial segment. *Cells* 9:1961. 10.3390/cells9091961 32858875 PMC7563147

[B20] Costa Verdera H. Gitz-Francois J. Schiffelers R. Vader P. (2017). Cellular uptake of extracellular vesicles is mediated by clathrin-independent endocytosis and macropinocytosis. *J. Control Release* 266 100–108. 28919558 10.1016/j.jconrel.2017.09.019

[B21] Cotrufo T. Andres R. Ros O. Perez-Branguli F. Muhaisen A. Fuschini G. (2012). Syntaxin 1 is required for DCC/Netrin-1-dependent chemoattraction of migrating neurons from the lower rhombic lip. *Eur. J. Neurosci.* 36 3152–3164. 10.1111/j.1460-9568.2012.08259.x 22946563

[B22] Cotrufo T. Perez-Branguli F. Muhaisen A. Ros O. Andres R. Baeriswyl T. (2011). A signaling mechanism coupling netrin-1/deleted in colorectal cancer chemoattraction to SNARE-mediated exocytosis in axonal growth cones. *J. Neurosci.* 31 14463–14480. 10.1523/JNEUROSCI.3018-11.2011 21994363 PMC6703395

[B23] De Vries M. Cooper H. (2008). Emerging roles for neogenin and its ligands in CNS development. *J. Neurochem.* 106 1483–1492. 10.1111/j.1471-4159.2008.05485.x 18485097

[B24] Fan J. Mansfield S. Redmond T. Gordon-Weeks P. Raper J. (1993). The organization of F-actin and microtubules in growth cones exposed to a brain-derived collapsing factor. *J. Cell Biol.* 121 867–878. 10.1083/jcb.121.4.867 8491778 PMC2119785

[B25] Ferreira A. Boucrot E. (2018). Mechanisms of carrier formation during clathrin-independent endocytosis. *Trends Cell Biol.* 28 188–200. 29241687 10.1016/j.tcb.2017.11.004

[B26] Fournier A. Nakamura F. Kawamoto S. Goshima Y. Kalb R. Strittmatter S. (2000). Semaphorin3A enhances endocytosis at sites of receptor-F-actin colocalization during growth cone collapse. *J. Cell Biol.* 149 411–422. 10.1083/jcb.149.2.411 10769032 PMC2175148

[B27] Fujiwara T. Mishima T. Kofuji T. Chiba T. Tanaka K. Yamamoto A. (2006). Analysis of knock-out mice to determine the role of HPC-1/syntaxin 1A in expressing synaptic plasticity. *J. Neurosci.* 26 5767–5776. 10.1523/JNEUROSCI.0289-06.2006 16723534 PMC6675267

[B28] Fuschini G. Cotrufo T. Ros O. Muhaisen A. Andres R. Comella J. (2018). Syntaxin-1/TI-VAMP SNAREs interact with Trk receptors and are required for neurotrophin-dependent outgrowth. *Oncotarget* 9 35922–35940. 10.18632/oncotarget.26307 30542508 PMC6267591

[B29] Gaffield M. Betz W. (2007). Synaptic vesicle mobility in mouse motor nerve terminals with and without synapsin. *J. Neurosci.* 27 13691–13700. 10.1523/JNEUROSCI.3910-07.2007 18077680 PMC6673622

[B30] Galas M. Chasserot-Golaz S. Dirrig-Grosch S. Bader M. (2000). Presence of dynamin–syntaxin complexes associated with secretory granules in adrenal chromaffin cells. *J. Neurochem.* 75 1511–1519. 10.1046/j.1471-4159.2000.0751511.x 10987831

[B31] Gallo G. Letourneau P. (2004). Regulation of growth cone actin filaments by guidance cues. *J. Neurobiol.* 58 92–102. 14598373 10.1002/neu.10282

[B32] Gerber S. Rah J. Min S. Liu X. de Wit H. Dulubova I. (2008). Conformational switch of syntaxin-1 controls synaptic vesicle fusion. *Science* 321 1507–1510. 18703708 10.1126/science.1163174 PMC3235364

[B33] Gil V. Del Rio J. (2019). Functions of plexins/neuropilins and their ligands during hippocampal development and neurodegeneration. *Cells* 8:206. 10.3390/cells8030206 30823454 PMC6468495

[B34] Ginty D. Segal R. (2002). Retrograde neurotrophin signaling: Trk-ing along the axon. *Curr. Opin. Neurobiol.* 12 268–274. 10.1016/s0959-4388(02)00326-4 12049932

[B35] Goshima Y. Nakamura F. Strittmatter P. Strittmatter S. (1995). Collapsin-induced growth cone collapse mediated by an intracellular protein related to UNC-33. *Nature* 376 509–514. 10.1038/376509a0 7637782

[B36] Guo D. Standley C. Bellve K. Fogarty K. Bao Z. (2012). Protein kinase Calpha and integrin-linked kinase mediate the negative axon guidance effects of Sonic hedgehog. *Mol. Cell. Neurosci.* 50 82–92. 10.1016/j.mcn.2012.03.008 22521536 PMC3383945

[B37] Hedgecock E. Culotti J. Hall D. (1990). The unc-5, unc-6, and unc-40 genes guide circumferential migrations of pioneer axons and mesodermal cells on the epidermis in *C. elegans*. *Neuron* 4 61–85. 10.1016/0896-6273(90)90444-k 2310575

[B38] Hernaiz-Llorens M. Rosello-Busquets C. Durisic N. Filip A. Ulloa F. Martinez-Marmol R. (2020). Growth cone repulsion to Netrin-1 depends on lipid raft microdomains enriched in UNC5 receptors. *Cell Mol. Life Sci.* 78 2797–2820. 10.1007/s00018-020-03663-z 33095273 PMC8004515

[B39] Hines J. Abu-Rub M. Henley J. (2010). Asymmetric endocytosis and remodeling of beta1-integrin adhesions during growth cone chemorepulsion by MAG. *Nat. Neurosci.* 13 829–837. 10.1038/nn.2554 20512137 PMC3133767

[B40] Hong K. Hinck L. Nishiyama M. Poo M. Tessier-Lavigne M. Stein E. (1999). A ligand-gated association between cytoplasmic domains of UNC5 and DCC family receptors converts netrin-induced growth cone attraction to repulsion. *Cell* 97 927–941. 10399920 10.1016/s0092-8674(00)80804-1

[B41] Igarashi M. Kozaki S. Terakawa S. Kawano S. Ide C. Komiya Y. (1996). Growth cone collapse and inhibition of neurite growth by Botulinum neurotoxin C1: A t-SNARE is involved in axonal growth. *J. Cell Biol.* 134 205–215. 10.1083/jcb.134.1.205 8698815 PMC2120926

[B42] Itofusa R. Tojima T. Kamiguchi H. (2017). Visualization of clathrin-mediated endocytosis during semaphorin-guided axonal growth. *Methods Mol. Biol.* 1493 287–298. 10.1007/978-1-4939-6448-2_21 27787859

[B43] Jahn R. Scheller R. (2006). SNAREs–engines for membrane fusion. *Nat. Rev.* 7 631–643. 10.1038/nrm2002 16912714

[B44] Jurney W. Gallo G. Letourneau P. McLoon S. (2002). Rac1-mediated endocytosis during ephrin-A2- and semaphorin 3A-induced growth cone collapse. *J. Neurosci.* 22 6019–6028. 10.1523/JNEUROSCI.22-14-06019.2002 12122063 PMC6757944

[B45] Kabayama H. Nakamura T. Takeuchi M. Iwasaki H. Taniguchi M. Tokushige N. (2009). Ca2^+^ induces macropinocytosis via F-actin depolymerization during growth cone collapse. *Mol. Cell. Neurosci.* 40 27–38. 10.1016/j.mcn.2008.08.009 18848894

[B46] Kabayama H. Takeuchi M. Taniguchi M. Tokushige N. Kozaki S. Mizutani A. (2011). Syntaxin 1B suppresses macropinocytosis and semaphorin 3A-induced growth cone collapse. *J. Neurosci.* 31 7357–7364. 10.1523/JNEUROSCI.2718-10.2011 21593320 PMC6622584

[B47] Keino-Masu K. Masu M. Hinck L. Leonardo E. D. Chan S. Culotti J. (1996). Deleted in colorectal cancer (DCC) encodes a netrin receptor. *Cell* 87 175–185. 8861902 10.1016/s0092-8674(00)81336-7

[B48] Keleman K. Dickson B. (2001). Short- and long-range repulsion by the *Drosophila* Unc5 netrin receptor. *Neuron* 32 605–617. 10.1016/s0896-6273(01)00505-0 11719202

[B49] Kim D. Ackerman S. (2011). The UNC5C netrin receptor regulates dorsal guidance of mouse hindbrain axons. *J. Neurosci.* 31 2167–2179. 10.1523/JNEUROSCI.5254-10.2011 21307253 PMC3073835

[B50] Kim T. Lee H. Seo I. Bae H. Suh D. Wu J. (2005). Netrin induces down-regulation of its receptor, deleted in colorectal cancer, through the ubiquitin-proteasome pathway in the embryonic cortical neuron. *J. Neurochem.* 95 1–8. 10.1111/j.1471-4159.2005.03314.x 16181408 PMC2683579

[B51] Kofuji T. Fujiwara T. Sanada M. Mishima T. Akagawa K. (2014). HPC-1/syntaxin 1A and syntaxin 1B play distinct roles in neuronal survival. *J. Neurochem.* 130 514–525. 10.1111/jnc.12722 24666284

[B52] Koivusalo M. Welch C. Hayashi H. Scott C. Kim M. Alexander T. (2010). Amiloride inhibits macropinocytosis by lowering submembranous pH and preventing Rac1 and Cdc42 signaling. *J. Cell Biol.* 188 547–563. 10.1083/jcb.200908086 20156964 PMC2828922

[B53] Kolpak A. Jiang J. Guo D. Standley C. Bellve K. Fogarty K. (2009). Negative guidance factor-induced macropinocytosis in the growth cone plays a critical role in repulsive axon turning. *J. Neurosci.* 29 10488–10498. 10.1523/JNEUROSCI.2355-09.2009 19710302 PMC2748960

[B54] Koo S. Markovic S. Puchkov D. Mahrenholz C. Beceren-Braun F. Maritzen T. (2011). SNARE motif-mediated sorting of synaptobrevin by the endocytic adaptors clathrin assembly lymphoid myeloid leukemia (CALM) and AP180 at synapses. *Proc. Natl. Acad. Sci. U. S. A.* 108 13540–13545. 10.1073/pnas.1107067108 21808019 PMC3158172

[B55] Lim J. Gleeson P. (2011). Macropinocytosis: An endocytic pathway for internalising large gulps. *Immunol. Cell Biol.* 89 836–843. 10.1038/icb.2011.20 21423264

[B56] Lin X. Mintern J. Gleeson P. (2020). Macropinocytosis in different cell types: Similarities and differences. *Membranes* 10:177. 10.3390/membranes10080177 PMC7463864 32756454

[B57] Luo Y. Raible D. Raper J. (1993). Collapsin: A protein in brain that induces the collapse and paralysis of neuronal growth cones. *Cell* 75 217–227. 10.1016/0092-8674(93)80064-l 8402908

[B58] Ly A. Nikolaev A. Suresh G. Zheng Y. Tessier-Lavigne M. Stein E. (2008). DSCAM is a netrin receptor that collaborates with DCC in mediating turning responses to netrin-1. *Cell* 133 1241–1254. 10.1016/j.cell.2008.05.030 18585357 PMC2491333

[B59] Matsumoto H. Nagashima M. (2010). Netrin-1 elevates the level and induces cluster formation of its receptor DCC at the surface of cortical axon shafts in an exocytosis-dependent manner. *Neurosci. Res.* 67 99–107. 10.1016/j.neures.2010.02.004 20170691

[B60] McCormick L. Gupton S. (2020). Mechanistic advances in axon pathfinding. *Curr. Opin. Cell. Biol.* 63 11–19. 31927278 10.1016/j.ceb.2019.12.003 PMC7247931

[B61] McKenna W. Wong-Staal C. Kim G. Macias H. Hinck L. Bartoe J. (2008). Netrin-1-independent adenosine A2b receptor activation regulates the response of axons to netrin-1 by controlling cell surface levels of UNC5A receptors. *J. Neurochem.* 104 1081–1090. 10.1111/j.1471-4159.2007.05040.x 17995930

[B62] Meier O. Boucke K. Hammer S. Keller S. Stidwill R. Hemmi S. (2002). Adenovirus triggers macropinocytosis and endosomal leakage together with its clathrin-mediated uptake. *J. Cell Biol.* 158 1119–1131. 10.1083/jcb.200112067 12221069 PMC2173207

[B63] Miller S. Sahlender D. Graham S. Honing S. Robinson M. Peden A. (2011). The molecular basis for the endocytosis of small R-SNAREs by the clathrin adaptor CALM. *Cell* 147 1118–1131. 10.1016/j.cell.2011.10.038 22118466 PMC3267021

[B64] Mishima T. Fujiwara T. Sanada M. Kofuji T. Kanai-Azuma M. Akagawa K. (2014). Syntaxin 1B, but not syntaxin 1A, is necessary for the regulation of synaptic vesicle exocytosis and of the readily releasable pool at central synapses. *PLoS One* 9:e90004. 10.1371/journal.pone.0090004 24587181 PMC3938564

[B65] Muramatsu R. Nakahara S. Ichikawa J. Watanabe K. Matsuki N. Koyama R. (2010). The ratio of ‘deleted in colorectal cancer’ to ‘uncoordinated-5A’ netrin-1 receptors on the growth cone regulates mossy fibre directionality. *Brain* 133 60–75. 10.1093/brain/awp266 19858080

[B66] O’Donnell M. Chance R. Bashaw G. (2009). Axon growth and guidance: Receptor regulation and signal transduction. *Annu. Rev. Neurosci.* 32 383–412. 19400716 10.1146/annurev.neuro.051508.135614 PMC4765433

[B67] Park S. Bin N. Sugita S. (2014). Novel role of glial syntaxin-1B in supporting neuronal survival. *J. Neurochem.* 130 469–471. 10.1111/jnc.12723 24750130

[B68] Pasterkamp R. Burk K. (2021). Axon guidance receptors: Endocytosis, trafficking and downstream signaling from endosomes. *Prog. Neurobiol.* 198:101916. 10.1016/j.pneurobio.2020.101916 32991957

[B69] Peng Y. He W. Tang J. Tao T. Chen C. Gao Y. (2010). Trio is a key guanine nucleotide exchange factor coordinating regulation of the migration and morphogenesis of granule cells in the developing cerebellum. *J. Biol. Chem.* 285 24834–24844. 10.1074/jbc.M109.096537 20516067 PMC2915719

[B70] Piper M. Anderson R. Dwivedy A. Weinl C. van Horck F. Leung K. (2006). Signaling mechanisms underlying Slit2-induced collapse of Xenopus retinal growth cones. *Neuron* 49 215–228. 10.1016/j.neuron.2005.12.008 16423696 PMC3689199

[B71] Poupon V. Polo S. Vecchi M. Martin G. Dautry-Varsat A. Cerf-Bensussan N. (2002). Differential nucleocytoplasmic trafficking between the related endocytic proteins Eps15 and Eps15R. *J. Biol. Chem.* 277 8941–8948. 10.1074/jbc.M108385200 11777906

[B72] Przyborski S. Knowles B. Ackerman S. (1998). Embryonic phenotype of Unc5h3 mutant mice suggests chemorepulsion during the formation of the rostral cerebellar boundary. *Development* 125 41–50. 10.1242/dev.125.1.41 9389662

[B73] Purohit A. Li W. Qu C. Dwyer T. Shao Q. Guan K. (2012). Down syndrome cell adhesion molecule (DSCAM) associates with uncoordinated-5C (UNC5C) in netrin-1-mediated growth cone collapse. *J. Biol. Chem.* 287 27126–27138. 10.1074/jbc.M112.340174 22685302 PMC3411055

[B74] Ros O. Barrecheguren P. Cotrufo T. Schaettin M. Rosello-Busquets C. Vilchez-Acosta A. (2018). A conserved role for Syntaxin-1 in pre- and post-commissural midline axonal guidance in fly, chick, and mouse. *PLoS Genet.* 14:e1007432. 10.1371/journal.pgen.1007432 29912942 PMC6029812

[B75] Ros O. Cotrufo T. Martinez-Marmol R. Soriano E. (2015). Regulation of patterned dynamics of local exocytosis in growth cones by netrin-1. *J. Neurosci.* 35 5156–5170. 10.1523/JNEUROSCI.0124-14.2015 25834042 PMC6705414

[B76] Rosello-Busquets C. de la Oliva N. Martinez-Marmol R. Hernaiz-Llorens M. Pascual M. Muhaisen A. (2019). Cholesterol depletion regulates axonal growth and enhances central and peripheral nerve regeneration. *Front. Cell. Neurosci.* 13:40. 10.3389/fncel.2019.00040 30809129 PMC6379282

[B77] Round J. Stein E. (2007). Netrin signaling leading to directed growth cone steering. *Curr. Opin. Neurobiol.* 17 15–21. 10.1016/j.conb.2007.01.003 17254765

[B78] Schiavo G. Matteoli M. Montecucco C. (2000). Neurotoxins affecting neuroexocytosis. *Physiol. Rev.* 80 717–766. 10747206 10.1152/physrev.2000.80.2.717

[B79] Schneider C. Rasband W. Eliceiri K. (2012). NIH Image to ImageJ: 25 years of image analysis. *Nat. Methods* 9 671–675. 22930834 10.1038/nmeth.2089 PMC5554542

[B80] Schutze S. Machleidt T. Adam D. Schwandner R. Wiegmann K. Kruse M. (1999). Inhibition of receptor internalization by monodansylcadaverine selectively blocks p55 tumor necrosis factor receptor death domain signaling. *J. Biol. Chem.* 274 10203–10212. 10.1074/jbc.274.15.10203 10187805

[B81] Singla B. Lin H. Ghoshal P. Cherian-Shaw M. Csanyi G. (2019). PKCdelta stimulates macropinocytosis via activation of SSH1-cofilin pathway. *Cell Signal.* 53 111–121. 10.1016/j.cellsig.2018.09.018 30261270 PMC6289628

[B82] Sundaramoorthy V. Walker A. Yerbury J. Soo K. Farg M. Hoang V. (2013). Extracellular wildtype and mutant SOD1 induces ER-Golgi pathology characteristic of amyotrophic lateral sclerosis in neuronal cells. *Cell Mol. Life Sci.* 70 4181–4195. 10.1007/s00018-013-1385-2 23765103 PMC11113712

[B83] Sundararajan L. Smith C. Watson J. Millis B. Tyska M. Miller D. (2019). Actin assembly and non-muscle myosin activity drive dendrite retraction in an UNC-6/Netrin dependent self-avoidance response. *PLoS Genet.* 15:e1008228. 10.1371/journal.pgen.1008228 31220078 PMC6605669

[B84] Swanson J. Watts C. (1995). Macropinocytosis. *Trends Cell Biol.* 5 424–428. 14732047 10.1016/s0962-8924(00)89101-1

[B85] Tian T. Zhu Y. Zhou Y. Liang G. Wang Y. Hu F. (2014). Exosome uptake through clathrin-mediated endocytosis and macropinocytosis and mediating miR-21 delivery. *J. Biol. Chem.* 289 22258–22267. 10.1074/jbc.M114.588046 24951588 PMC4139237

[B86] Tojima T. Akiyama H. Itofusa R. Li Y. Katayama H. Miyawaki A. (2007). Attractive axon guidance involves asymmetric membrane transport and exocytosis in the growth cone. *Nat. Neurosci.* 10 58–66. 17159991 10.1038/nn1814

[B87] Tojima T. Itofusa R. Kamiguchi H. (2010). Asymmetric clathrin-mediated endocytosis drives repulsive growth cone guidance. *Neuron* 66 370–377. 20471350 10.1016/j.neuron.2010.04.007

[B88] Urbina F. Gupton S. L. (2020). SNARE-Mediated Exocytosis. *Front. Mol. Neurosci.* 13:133. 10.3389/fnmol.2020.00133 32848598 PMC7427632

[B89] Valdez G. Akmentin W. Philippidou P. Kuruvilla R. Ginty D. Halegoua S. (2005). Pincher-mediated macroendocytosis underlies retrograde signaling by neurotrophin receptors. *J. Neurosci.* 25 5236–5247. 10.1523/JNEUROSCI.5104-04.2005 15917464 PMC6724820

[B90] Williams M. Wu S. McKenna W. Hinck L. (2003). Surface expression of the netrin receptor UNC5H1 is regulated through a protein kinase C-interacting protein/protein kinase-dependent mechanism. *J. Neurosci.* 23 11279–11288. 10.1523/JNEUROSCI.23-36-11279.2003 14672991 PMC6740510

[B91] Wu Y. Tejero R. Arancillo M. Vardar G. Korotkova T. Kintscher M. (2015). Syntaxin 1B is important for mouse postnatal survival and proper synaptic function at the mouse neuromuscular junctions. *J. Neurophysiol.* 114 2404–2417. 10.1152/jn.00577.2015 26203110 PMC4620129

[B92] Xu J. Luo F. Zhang Z. Xue L. Wu X. Chiang H. (2013). SNARE proteins synaptobrevin. SNAP-25, and syntaxin are involved in rapid and slow endocytosis at synapses. *Cell Rep.* 3 1414–1421. 10.1016/j.celrep.2013.03.010 23643538 PMC3672373

[B93] Zeineddine R. Yerbury J. (2015). The role of macropinocytosis in the propagation of protein aggregation associated with neurodegenerative diseases. *Front. Physiol.* 6:277. 10.3389/fphys.2015.00277 26528186 PMC4607857

[B94] Zhang Z. Wang D. Sun T. Xu J. Chiang H. Shin W. (2013). The SNARE proteins SNAP25 and synaptobrevin are involved in endocytosis at hippocampal synapses. *J. Neurosci.* 33 9169–9175. 23699527 10.1523/JNEUROSCI.0301-13.2013 PMC3692273

[B95] Zhou F. Cohan C. (2001). Growth cone collapse through coincident loss of actin bundles and leading edge actin without actin depolymerization. *J. Cell Biol.* 153 1071–1084. 10.1083/jcb.153.5.1071 11381091 PMC2174321

[B96] Zylbersztejn K. Petkovic M. Burgo A. Deck M. Garel S. Marcos S. (2012). The vesicular SNARE Synaptobrevin is required for Semaphorin 3A axonal repulsion. *J. Cell Biol.* 196 37–46. 10.1083/jcb.201106113 22213797 PMC3255983

