# Supplementary material for: Syntaxin-1 is necessary for UNC5A-C/Netrin-1-dependent macropinocytosis and chemorepulsion
Source: Front Mol Neurosci. 2023 Sep 27;16:1253954. doi: 10.3389/fnmol.2023.1253954 (PMC10565356; doi:10.3389/fnmol.2023.1253954)
Supplement: Supplementary file 1 [file Presentation_1.pdf]

Supplementary Material

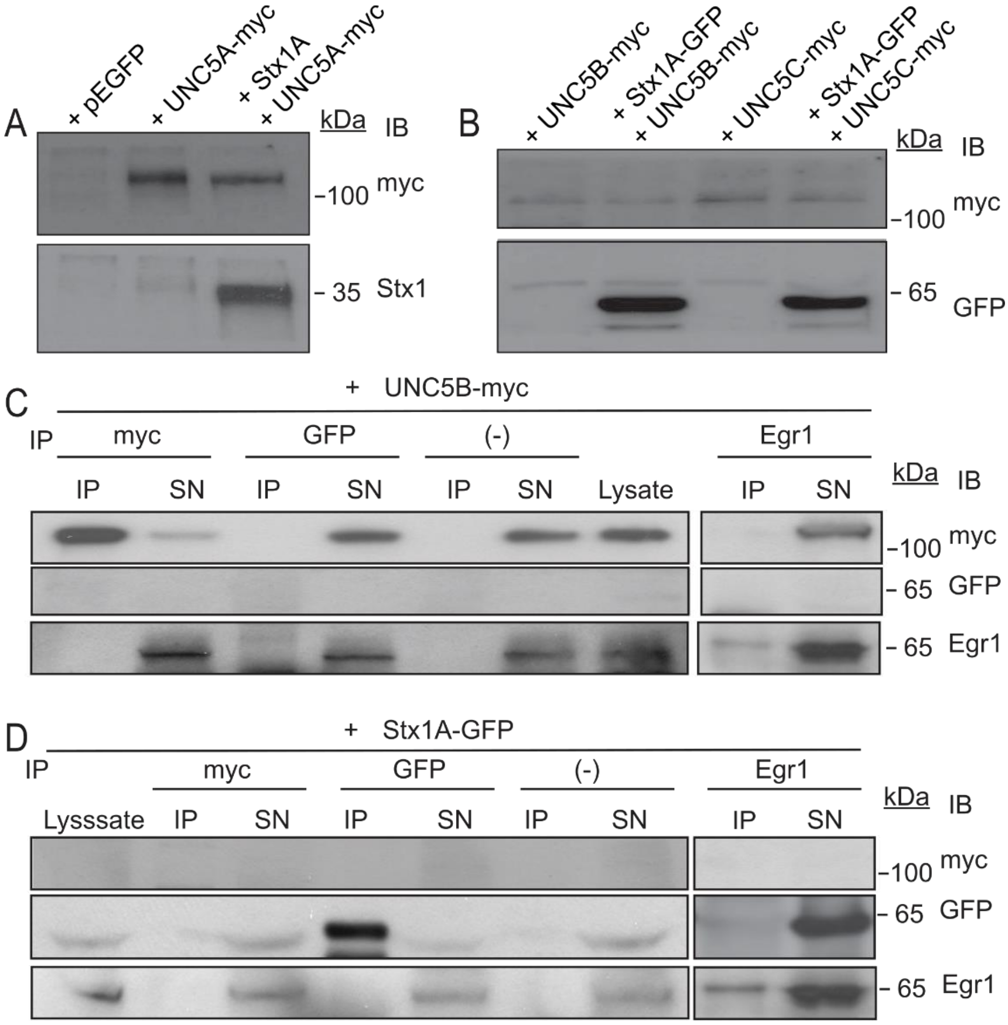

Figure S1. **Loading controls and IP controls for blots presented in Fig. 1. A, B** HEK-293T cells were transfected with the indicated combination of plasmids (pEGFP, UNC5A-myc, both UNC5A-myc and Stx1A, UNC5B-myc, both Stx1A-GFP and UNC5B-myc, UNC5C-myc, both Stx1A-GFP and UNC5C-myc). Equal amounts of protein lysates were loaded and detected by immunoblotting (IB) using anti-Stx1 (Stx1), anti-myc (myc) or anti-GFP (GFP) antibodies. **C, D** HEK-293T cells were transfected with UNC5B-myc (C) or with Stx1A-GFP (D). Protein lysates were immunoprecipitated (IP) with anti-myc, anti-GFP, anti-Egr1 (Egr1) or without antibodies (-). Anti-Egr1 is used as a non-interacting unspecific control, and the absence of antibodies as a negative control. Immunoprecipitation of UNC5B, Stx1A and Egr1 was detected by immunoblotting (IB) using anti-GFP, anti-myc or anti-Egr1 antibodies. Only anti-myc antibody was able to immunoprecipitate UNC5B-myc and could not immunoprecipitate Stx1-GFP or Egr1 (**C**). Only anti-GFP antibody was able to immunoprecipitated Stx1-GFP and could not immunoprecipitated UNC5B-myc or Egr1 (D). In the absence of antibodies (-) or using anti-Egr1 we could not immunoprecipitate UNC5B-myc or Stx1-GFP.

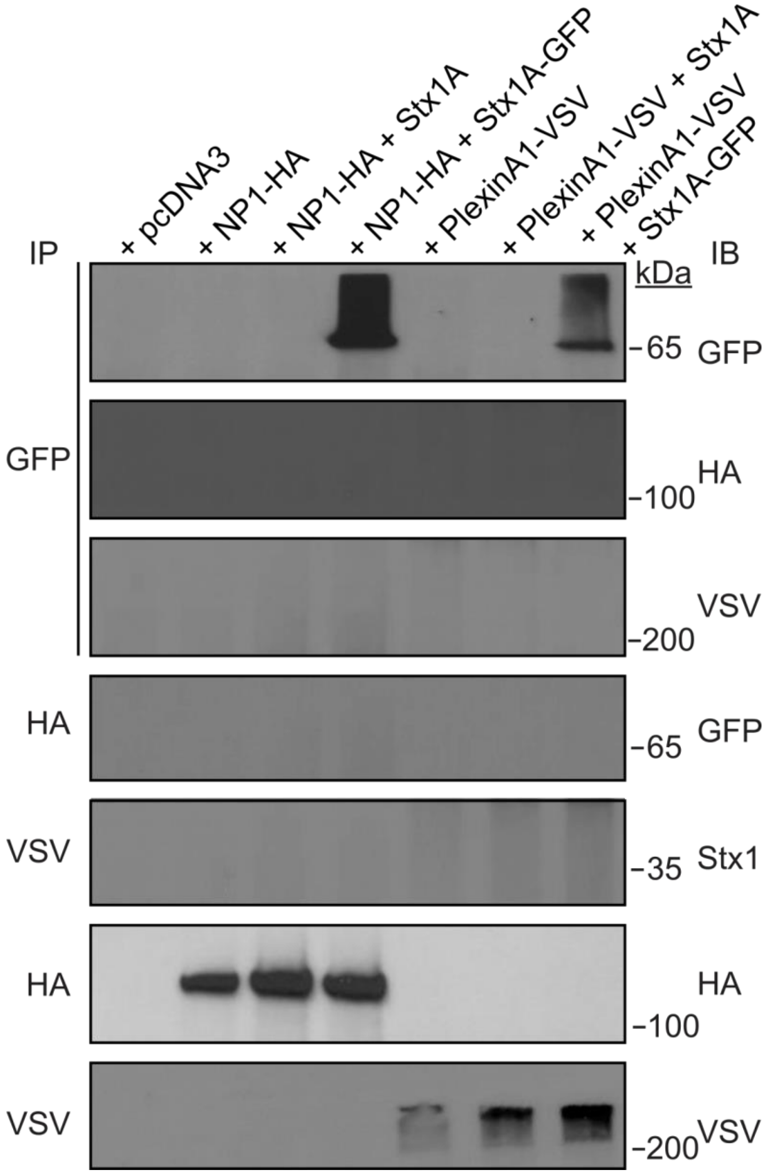

Figure S2. Stx1 does not interact with Semaphorin receptors. HEK-293T cells were transfected with the indicated combination of plasmids (empty vector pcDNA3, Neuropilin-1-HA (NP1-HA), NP1-HA and Stx1A, NP1-HA and Stx1A-GFP, PlexinA1-VSV, PlexinA1-VSV and Stx1A, or PlexinA1-VSV and Stx1A-GFP). Protein lysates were immunoprecipitated (IP) with anti-GFP, anti-HA or anti-VSV antibodies. Co-immunoprecipitation was detected by immunoblotting (IB) using anti-GFP (to detect Stx1A), anti-HA (to detect NP1) or anti-VSV (to detect PlexinA1) antibodies.

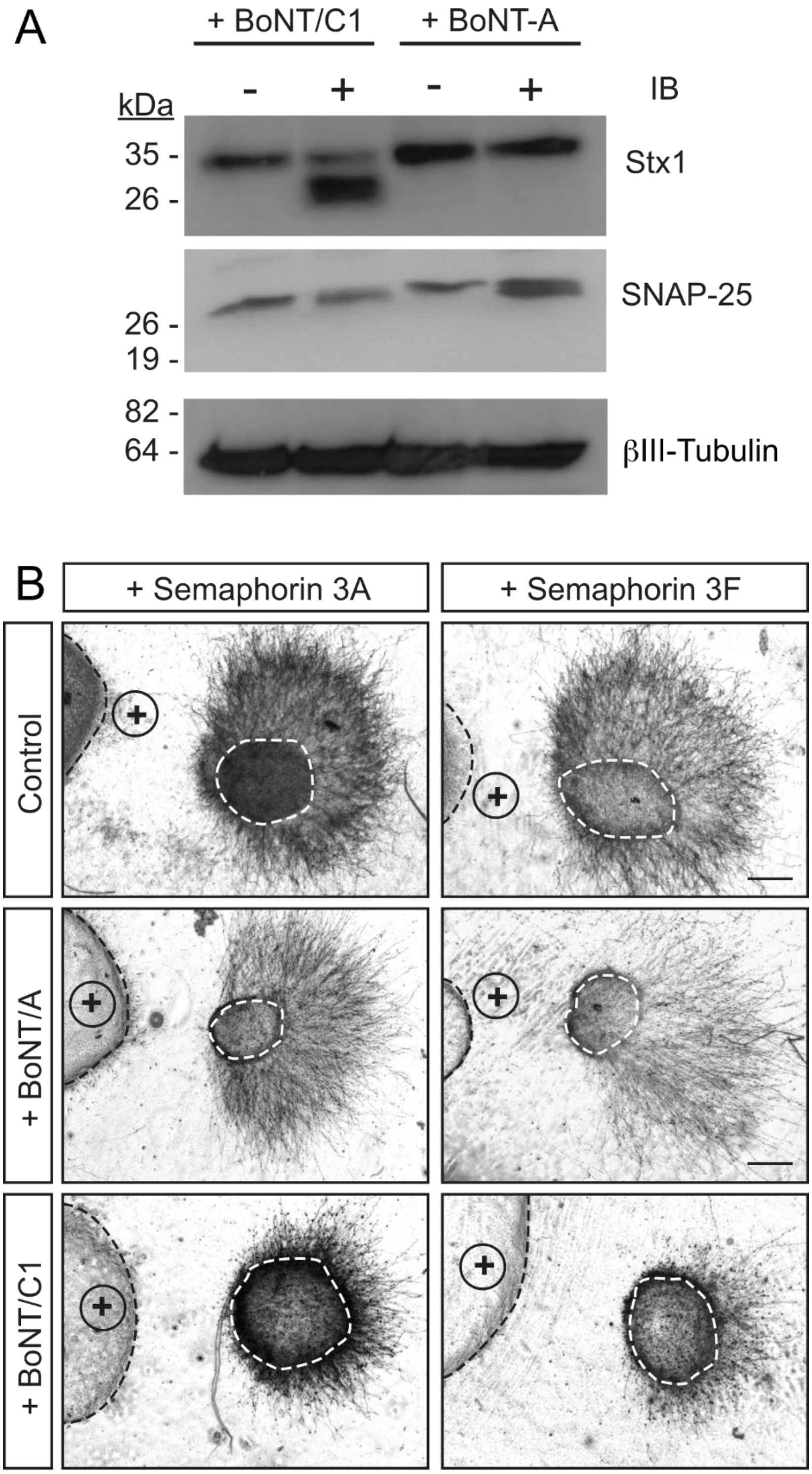

Figure S3. BoNT/C1 cleaves specifically Stx1 from EGL neurons, but does not affect the Semaphorin-mediated hippocampal repulsion. A Lysates from untreated EGL neurons or those treated with BoNT/C1 (15 nM) or BoNT/A (25 nM) for 45 min were immunoblotted against anti-Stx1, anti-SNAP-25 or anti-βIII-tubulin. **B** Representative images of hippocampal explants from E16 mice, immunodetected with anti-βIII-tubulin. Explants were confronted with Semaphorin 3A- or Semaphorin 3F-secreting aggregates. Explants were cultured in the absence of BoNTs (control) or in medium supplemented with 25 nM BoNT/A (+ BoNT/A) or 15 nM BoNT/C1 (BoNT/C1). HEK-293T aggregates are outlined with a dashed black line, explants are outlined with a dashed white line. Scale bars represent 100 µm.

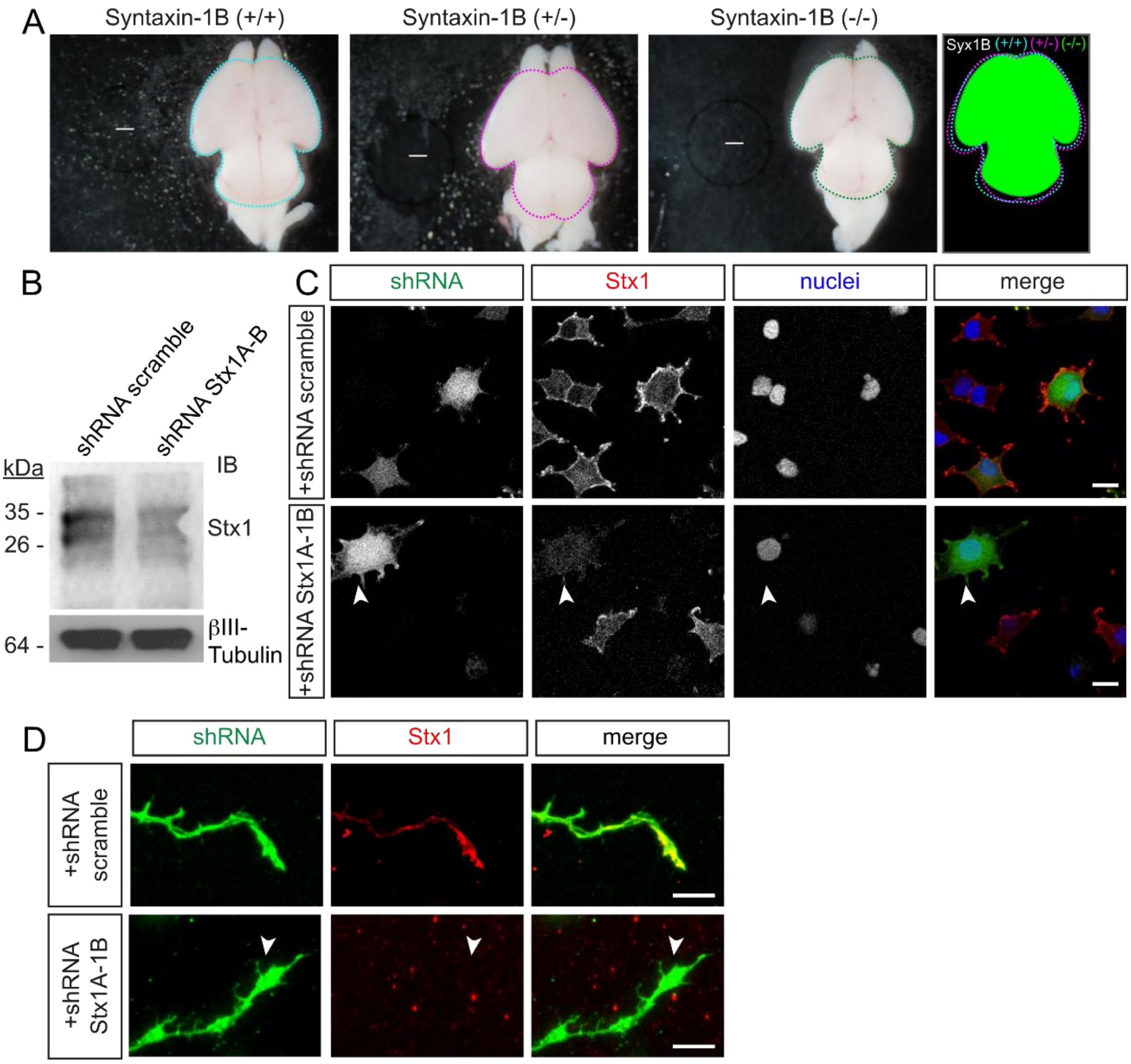

Figure S4. Cleavage and downregulation of Stx1. A Representative images of brains from embryonic E17 mice with different genetic backgrounds (Stx1B (+/+) -cyan-, Stx1B (+/-) -magenta- and Stx1B (-/-) -green-), together with their overlapping silhouettes to compare their size. **B** Western blot of lysates from PC12 cells transfected with a shRNA scrambled control plasmid, or with a shRNA plasmid against both Stx1A and Stx1B (shRNA Stx1A-1B). Samples were subjected to urea/SDS-PAGE, identifying two Stx1 bands corresponding to Stx1B (upper band) and Stx1A (lower band). **C** Representative images of PC12 cells transfected with a shRNA scrambled control plasmid, or with a shRNA plasmid against both Stx1A and Stx1B (shRNA Stx1A-1B), and immunostained with anti-GFP to identify cells transfected with the shRNA (shRNA), with anti-Stx1 and with DAPI. Note that Stx1A-1B shRNA-transfected cells (arrowheads) display no Stx1 immunolabelling. **D.** Representative images of EGL neurons transfected with a shRNA scrambled control plasmid, or with a shRNA plasmid against both Stx1A and Stx1B (shRNA Stx1A-1B), and immunostained with anti-GFP to detect neurons transfected with the shRNA (shRNA), with anti-Stx1 and with DAPI. Note that Stx1A-1B shRNA-transfected neurons (arrowheads) display no Stx1 immunolabelling. Scale bar represents 10 μm.
